# The implementation of colorectal cancer screening interventions in low-and middle-income countries: a scoping review

**DOI:** 10.1186/s12885-021-08809-1

**Published:** 2021-10-19

**Authors:** Désirée Schliemann, Kogila Ramanathan, Nicholas Matovu, Ciaran O’Neill, Frank Kee, Tin Tin Su, Michael Donnelly

**Affiliations:** 1 Centre for Public Health and UKCRC Centre of Excellence for Public Health, Queen’s University Belfast, Belfast, UK; 2 Global Public Health, Jeffrey Cheah School of Medicine and Health Sciences, Monash University Malaysia, Subang Jaya, Selangor Malaysia; 3 South East Asia Community Observatory (SEACO), Jeffrey Cheah School of Medicine and Health Sciences, Monash University Malaysia, Subang Jaya, Selangor Malaysia

**Keywords:** Bowel cancer, Colorectal cancer, Screening, LMIC, Review, Implementation

## Abstract

**Background:**

Low- and middle-income countries (LMICs) experienced increasing rates of colorectal cancer (CRC) incidence in the last decade and lower 5-year survival rates compared to high-income countries (HICs) where the implementation of screening and treatment services have advanced. This review scoped and mapped the literature regarding the content, implementation and uptake of CRC screening interventions as well as opportunities and challenges for the implementation of CRC screening interventions in LMICs.

**Methods:**

We systematically followed a five-step scoping review framework to identify and review relevant literature about CRC screening in LMICs, written in the English language before February 2020. We searched Medline, Embase, Web of Science and Google Scholar for studies targeting the general, asymptomatic, at-risk adult population. The TIDieR tool and an implementation checklist were used to extract data from empirical studies; and we extracted data-informed insights from policy reviews and commentaries.

**Results:**

CRC screening interventions (*n* = 24 studies) were implemented in nine middle-income countries. Population-based screening programmes (*n* = 11) as well as small-scale screening interventions (*n* = 13) utilised various recruitment strategies. Interventions that recruited participants face-to-face (alone or in combination with other recruitment strategies) (10/15), opportunistic clinic-based screening interventions (5/6) and educational interventions combined with screening (3/4), seemed to be the strategies that consistently achieved an uptake of > 65% in LMICs. FOBT/FIT and colonoscopy uptake ranged between 14 and 100%. The most commonly reported implementation indicator was ‘uptake/reach’. There was an absence of detail regarding implementation indicators and there is a need to improve reporting practice in order to disseminate learning about how to implement programmes.

**Conclusion:**

Opportunities and challenges for the implementation of CRC screening programmes were related to the reporting of CRC cases and screening, cost-effective screening methods, knowledge about CRC and screening, staff resources and training, infrastructure of the health care system, financial resources, public health campaigns, policy commitment from governments, patient navigation, planning of screening programmes and quality assurance.

**Supplementary Information:**

The online version contains supplementary material available at 10.1186/s12885-021-08809-1.

## Background

Colorectal cancer (CRC) constitutes a serious global health burden, affecting nations across all gross national income per capita groupings [1]. It is the fourth most common cancer (age-standardised incidence rate: 19.7/100,000) and third most common cause of cancer deaths (age-standardised mortality rate: 9/100,000) worldwide. Reported CRC age-standardised incidence rate in high-income countries (HICs; 24.3/100,000)) was four-times higher than in low-and middle-income countries (LMICs; 6.4/100,000) whilst mortality rates (age-standardised mortality rate: 10.6/100,000 and 3.9/100,000, respectively) were 17% higher in LMICs in 2018 [1]. HICs have implemented population-based CRC screening and these programmes have demonstrated a reduction in CRC mortality between 8 and 52% [2–4]. Due to the increased chances of survival of CRC when detected at an early stage, the International Agency for Cancer Research recommends biannual CRC screening in countries where follow-up treatment is accessible [5].

The most commonly recommended and implemented tests for population-based CRC screening are stool tests, i.e. the guaiac faecal occult blood test (FOBT/gFOBT) and the faecal immunochemical test (FIT/iFOBT) [6, 7], which are designed to detect small amounts of blood in stool samples of asymptomatic, average-risk individuals. Positive FOBT or FIT results require follow-up with invasive, visual screening techniques (i.e. colonoscopies or sigmoidoscopies) to confirm diagnosis [6, 7]. HICs tend to offer population-based screening programmes and invite everyone aged ≥50 years to send stool samples through the post – an approach that has been shown to be effective in increasing CRC screening uptake [8]. However, this approach is not feasible in LMICs due to a lack of infrastructure and resources. CRC-testing in LMICs tends to be unavailable or opportunistic in delivery and, often, CRC cases are diagnosed when they are symptomatic. Discrepancies between high CRC mortality rates and absence or lack of screening in LMICs compared to HICs could potentially be addressed through screening and adequate follow-up treatment. A systematic, system-strengthening approach is required to address differences between LMICs and HICs in terms of health care organisation and delivery, resources, infrastructure and social norms and to engage eligible screening populations [9–11]. Screening interventions need to be designed and delivered with these complex implementation considerations in mind [9, 12]. Therefore, the aim of this review was to scope the literature regarding a) the content, implementation and uptake of CRC screening interventions/ programmes in LMICs and b) opportunities and challenges in terms of the factors that facilitate and inhibit the implementation of CRC screening interventions/programmes in LMICs.

## Methods

The scoping review was guided by Arksey and O’Malley’s five-step framework (i.e. identifying the research question; identifying relevant studies; study selection; charting the data; collating and reporting the results) [13]. The review protocol was developed and published prior to the search [14]. A scoping review was deemed the most appropriate method to ensure that we captured and analysed the full range and breadth of studies about CRC screening in LMICs. We followed the Preferred Reporting Items for Systematic Reviews and Meta-Analysis: extension for scoping reviews (PRISMA-ScR) guidelines for reporting the results (Supplementary Table **1**) [15]. In this review we distinguished between a) empirical studies of CRC screening interventions or programmes that were designed explicitly to encourage the use of CRC screening (from here on referred to as ‘interventions’) and b) commentaries/editorials and policy reviews that presented views regarding the implementation of CRC screening interventions in LMICs and related opportunities and challenges. Search criteria evolved from review team discussions and with guidance from an experienced subject librarian. MeSH terms related to three key concepts: ‘colorectal cancer’, ‘screening’ and ‘LMICs’. Filters were applied to select human and English language studies only. Studies were included if they were a) written in the English language before 27th January 2020 (date of search); b) set in/focused on LMICs (as defined by the World Bank based on gross national income per capita in 2018, **Supplementary Table** **2**); c) targeted at the general adult population; and d) designed for asymptomatic populations ‘at-risk’ of CRC (i.e. people aged ≥40 or 50 years or with a history of CRC). Studies aimed at improving cancer screening amongst cancer patients and health care professionals were excluded from this review, as were intervention protocols if they did not contain information about implementation. Searches were conducted in MEDLINE (**Supplementary Table** **3**), EMBASE, Web of Science and Google Scholar and we searched reference lists of relevant studies and reviews as well as Google Web for unpublished reports. The title and abstract screens were conducted by D.S. and D.S. and K.R. or N.M. conducted, independently, the full-text screen and data extraction. Data from empirical studies were extracted according to a) an adapted ten-item version of the Template for Intervention Description and Replication (TIDieR) checklist for reporting interventions (**Supplementary Table** **4**) and b) a template developed by Tierney et al. [16] to describe implementation considerations. Studies were categorised into three groups according to the percentage uptake of the FIT/FOBT: < 45%, 45–65 and > 65% based on the European CRC screening guidelines that recommend a target uptake of > 65% and that the lowest acceptable uptake is 45% [17]. Data from commentaries were extracted qualitatively into two major categories in NVIVO version 12.0, i.e. opportunities and challenges for implementation of CRC screening uptake. The ten most commonly reported opportunities and challenges were summarised and charted.

## Results

**Fig. 1:**
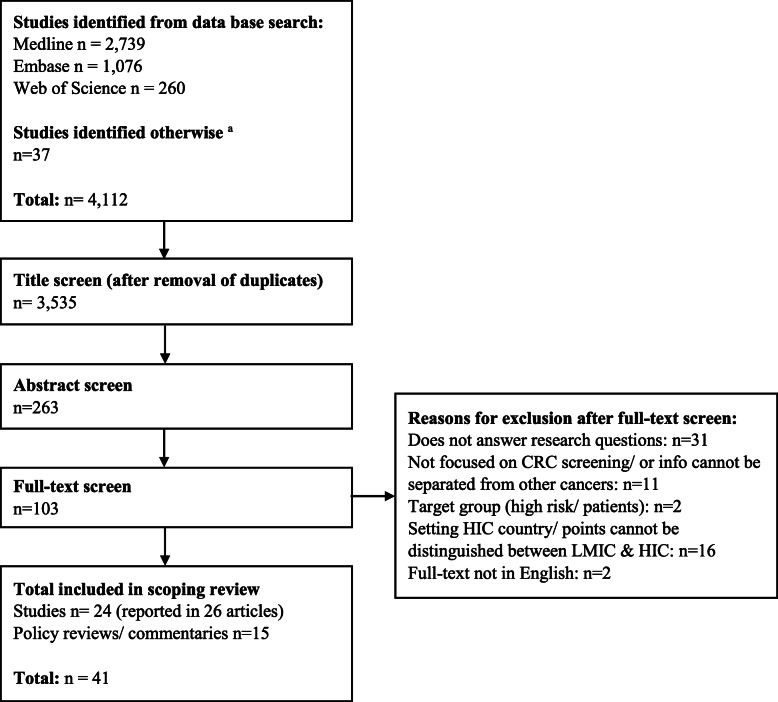
Number of studies identified through literature search. CRC – colorectal cancer; HIC – high-income countries; LMIC – low- and middle-income country. ^a^ includes google scholar, google web, contacting search of reference lists, contacting experts who work in the field for references

The search generated 4112 articles (Fig. 1) of which 41 were included in this scoping review (26 manuscripts of 24 studies and 15 policy reviews/commentaries). The findings from the studies and commentaries are described separately.

The full results of the data extraction according to the TIDieR checklist are presented in **Supplementary Table** **4** and a condensed version with key information about interventions is presented in Table 1. All studies were implemented in upper-middle income countries, mostly in Asia (9 in China, 3 in Malaysia and 2 in Thailand), followed by Europe (1 in Bulgaria, 1 in Romania and 2 in Serbia), the Middle East (1 in Jordan and 3 in Iran) and 2 in Mexico. Studies recruited between 197 and 1,381,561 participants and took place between 1989 and 2017. The duration of reported study periods was between 3.5 months and 7 years.

**Table 1:** Overview of included studies

Study information Country	Screening procedure			Recruitment & sampling							Reminder	Education provided as part of intervention	Intervention timeframe	Participants	Screening uptake/ participation (%)		
	HRFQ/ RA	FOBT/FIT	Colonoscopy^a^	Who recruited participants	Sampling	Face-to-face	Phone	Letter/ e-mail	Media	Where recruited					HRFQ	FOBT/ FIT	Colonoscopy
**Stool test uptake > 65%**																	
*Cai* et al *2011* *Ma* et al *2012* *Cai* et al *2016* [1–3] **China**	x	2 x FIT 1 x FOBT	If +	*Unclear* *(likely led by physician)*	Population-based	x	–	–	–	C	–	–	2007–2009	Residents aged 40–74 y. *(medically & economically underserved)* Enrolled *n* = 31,963	84.6	76.2 (1 x FIT) 65.3 (2 x FIT)	78.7
Gong et al. 2018 [5] **China**	x	2 x FIT	If +	CHC staff	Population -based	(x)	–	–	x	C	x	–	Jan – Dec 2013	Residents aged 50–74 y. Registered *n* = 809,528	97.7	97.7	39.8
*Zheng* et al *2003* [10] **China**	x	1 x RPHA- FOBT	Sigmoidoscopy if +	Field interviewers	Population-based	x	–	–	–	C	–	–	Data used from 1989 to 1996	Residents aged ≥30 y. Recruited *n* = 75,813/192,261 eligible residents (39.4%) RPHA-FOBT & Risk assessment: 82.7%	82.7	82.7	73.6
Hassan et al. 2016 [12] **Malaysia**	–	1 x FIT, 2nd if negative)	If +	Physician	Purposive sampling	x	–	–	–	CHC/ H	–	–	2013	Patients who underwent iFOBT in 2013 aged ≥50 y. Enrolled *n* = 750	–	R1: 94.7 R2: 90.6	68.1
Noriah et al. 2010 [13] **Malaysia**	–	1 x FOBT	If +	Health care workers/ media	IG1: Random sampling IG2: Voluntary Response sampling IG3: Convenience sampling	IG1 & IG 3	–	–	IG2	C CHC	–	–	15th Sept – 31st Dec 2007	Adults aged ≥50 y. 605/2574 participants IG1 & IG 2: residents IG3: patients IG1 *n* = 151 (86.6%) IG2 *n* = 275 (13.8%) IG3 *n* = 179 (44.8%)	–	IG1: 95.4 IG2: 87.6 IG3: 92.2	*Unclear*
Tze et al. 2016 [14] **Malaysia**	–	1 x FIT	If +	Volunteer -medical students (with support from community leaders)	Convenience sampling	x	–	–	–	C	–	Awareness Workshops (group)	2010–2015 *(1-y project in 5 different district every year)*	Residents aged ≥50 y. 1581 FIT kits were distributed	–	80–100% (varied by year)	63.2–78.6
Aniwan et al. 2017 [16] **Thailand**	–	1 x FIT	1 x	*Unclear* *(likely led by nurses)*	Convenience sampling	x	–	–	–	H	–	–	Dec 2014 – Dec 2016	Participants from 6 hospitals across Thailand aged 50–75 y. Enrolled *n* = 1740	–	98.4	98.4
Remes-Troche et al. 2020 [18] **Mexico**	–	1 x FIT	If +	Media (*unclear by whom)*	Voluntary response sampling	–	–	–	x	C	–	–	15 May 2015–15 Jan 2016 (Ads for 3 months)	Adults aged ≥50 y. Reply to ads *n* = 502 Eligible *n* = 473	–	85.8	87.5
Dimova et al. 2015 [19] **Bulgaria**	–	1 x FIT (& 1 if +)	Fibro-C if +	Physicians *(contacted people at home)*	Purposive sampling	–	x	x	–	C	x	–	Jun – Sept 2013	Health-insured, asymptomatic adults aged ≥45 y. Invited *n* = 600	–	78.8	75
Sucevaeanu et al. 2005 [20] **Romania**	–	1 x FOBT *3 samples requested*	If +	Media *(unclear by whom)*	Voluntary response sampling	–	–	–	x	C	–	–	May 2003 – Nov 2004	Adults aged ≥50 y. Patients interested *n* = 1769	–	70.3	92.6
Scepanovic et al. 2017 [22] **Serbia**	–	1 x FIT	If +	Physicians	Random sampling	x	–	–	–	CHC	–	–	Aug – Nov 2013	Adults aged 50–74 y. Invited n = 50,894	–	67.8	69.7
Gholampour et al. 2018 [24] **Iran**	–	1 x FOBT	If +	*Unclear*	Convenience sampling	(x)	–	–	–	CHC	x	8 x session (group)	2016–2017	Males aged > 50 y. Participants *n* = 200	–	IG: 74.0 CG: 6.0	100 (n = 1)
Salimzadeh et al. 2017 [25] **Iran**	–	1 x FIT	If +	Health navigators	Purposive sampling	x	x	–	x	C	x	1 x session (individual)	*Unclear*	Adults aged 45–75 y. Invited *n* = 1438	–	96.0	60.0
**Stool test uptake 45–65%**																	
Khuhaprema et al. 2014 [15] **Thailand**	–	1 x FIT	If +	CHW	Population-based	x	–	–	–	C	–	–	April 2011- Nov 2012	Residents aged 50–65 y. Invited *n* = 127,301	–	62.9	71.8
Bankovic Lazarevic et al. 2016 [21] **Serbia**	–	1 x FIT	If +	Physicians	Population- based	–	x	x	–	C	–	–	2013–2014 (2 years)	Adults aged 50–74 y. Invited *n* = 99,595	–	62.5	42.1
Huang et al. 2014 [9] **China**	x	1 x FOBT vs. 1 x FOBT & HRFQ	If +	CDC officials	Population- based	x	–	–	–	C	–	–	July 2006 – Dec 2008	Residents aged 40–74 y. Approached *n* = 400,000 *(unclear how many participated)*	53.2	45.4 vs 53.2	37.3 vs. 46.8
**Stool test uptake < 45%**																	
Wu et al. 2019 [7] **China**	x	2 x FIT	If +	*Unclear, author refers to community mobilization]*	Population- based	(x)	–	–	–	C	–	–	2 rounds (2013–2017)	Residents aged 50–79 y. Eligible n = 1,356,068	39.7	39.7	23.5
Abuadas et al. 2018 [23] **Jordan**	–	Suggested FOBT		Researchers	Convenience sampling	x	–	–	–	H	–	1 x 1-h session (group)	1st July – 3rd Nov 2015	Adults aged 50–75 y. Participants *n* = 197	–	IG: 35.7 CG: 8.1	–
*Li, Qian* et al *2019* [6] **China**	x	1 x FOBT	If +	Physician	Population- based	–	–	x	–	C	x	–	2 rounds (2013–2016)	Residents with medical insurance aged 50–74 y. Invited n = 1,262,214	35.2	35.2	26.3
Salimzadeh et al. 2013 [26] **Iran**	–	Suggested FOBT	–	Research assistants	Convenience sampling	–	x	–	–	C (Health clubs)	x	1 x 20-min Session (unclear)	July 2011-Nov 2012	Adults aged ≥50 y. *n* = 360	–	FOBT IG: 26.0 CG: 2.8	IG: 5.0 CG: 0
Huang et al. 2011 [8] **China**	–	1 x FOBT	–	Health workers	Cluster random sampling	x	–	–	–	C	–	Monthly lectures (group)	May 2008 – May 2010	Residents Person-times attending lectures *n* = 8981 Survey completed *n* = 1041	–	24.5	12
Lin et al. 2019 [11] **China**	x	2 x FIT	If +	Media/ SMS *(unclear who sent)*	Population-based	–	–	–	x	C	x	–	2015–2017	Residents aged 50–74 y. 350,581/2,283,214 residents completed 1st stage of screening	15.4	14.0	18.9
**Colonoscopy only**																	
Garcia-Osogobio et al. 2015 [17] **Mexico**	–	–	1x	Employer	Convenience sampling	–	–	x	x	WP (H)	–	–	2009–2010	Employees aged 40–79 y. Invited n = 600	–	–	16.5
Chen et al. 2019 [4] **China**	x	–	If +	Trained staff	Population- based	x	x	–	x	C	–	–	October 2012–October 2015	Residents aged 40–69 y. Recruited n = 1,381,561 High-risk *n* = 182,927	*NR*	–	14.0

^a^ Colonoscopy attendance: % describes colonoscopy attendance of those with a positive FOBT/FIT/HRFQ (except for interventions where colonoscopy was the primary screening tool)

C – community, CG – control group, CHC – community health clinics/ centres; CHW – community health worker; CRC – colorectal cancer; FOBT – Fecal Occult Blood Test; iFOBT/ FIT – Fecal Immunochemical Test; GP – general practitioner; H – hospital, HN – health navigator; HRFQ – high risk factor questionnaire; HW – health worker; IG – intervention group, NR – not reported; RA – risk assessment; WP-workplace, y – years

(x) not clearly stated but assumption made by authors based on information provided

**Sampling methods:**

Voluntary response sampling – participants were self-chosen

Convenience sampling – data was collected from conveniently available participants

Population-based sampling – all eligible individuals of a defined population were invited

Purposive sampling – participants were purposively selected to represent the target population

**Fig. 2:**
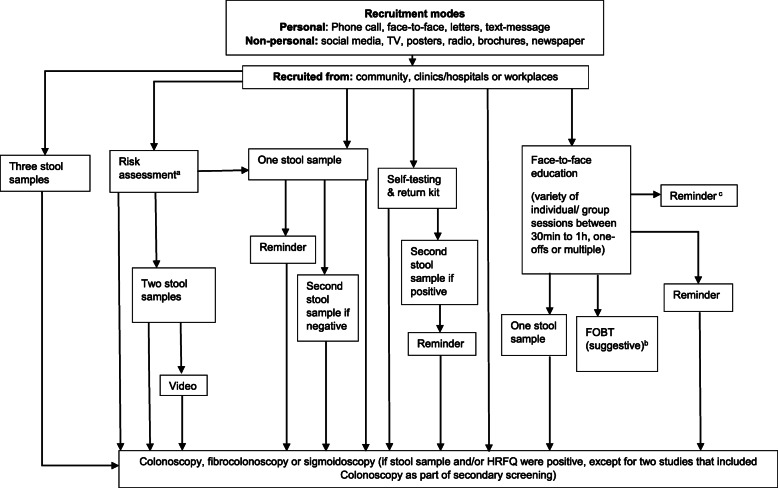
Mapping of interventions*.* This diagram presents the flow of all CRC screening interventions presented in this scoping review. Participants were recruited from either the community (population-level or small scale), clinics or workplaces through one of the recruitment modes described. Participants were then mostly counselled/ informed about the intervention and asked to either collect stool samples (for FIT/ FOBT), complete risk assessments, participate in educational session or colonoscopy/ sigmoidososcopy/ fibrocolonoscopy or a combination of those. Intervention details are described in **Supplementary Materials****3**. ^a^Risk assessments were either described as ‘risk assessment’, high-risk factor questionnaire (HRFQ) or the Asian Pacific Risk Score was applied. ^b^FOBT suggestive – only after educational interventions, participants were encouraged to do screening and either given a stool kit or not (based on self-report rather than clinical intervention). ^c^ Participants received education and were given reminders to complete CRC screening, however, screening was not offered as part of the intervention

**Fig. 3:**
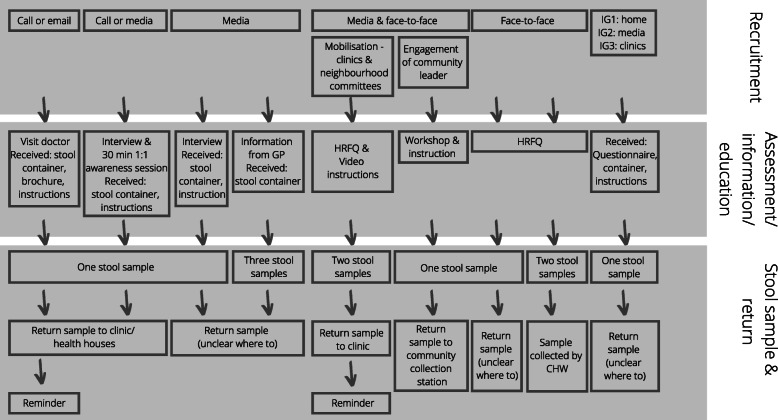
Overview of interventions that recruited participant from their homes & public places and achieved a stool test uptake of > 65%

**Fig. 4:**
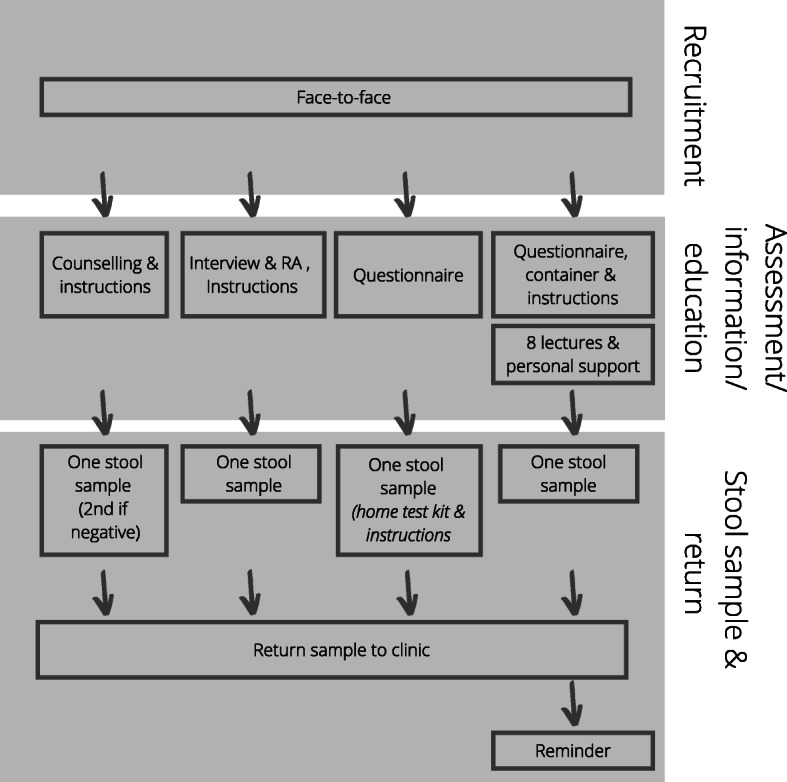
Overview of interventions that recruited participants from clinics and achieved a stool test uptake of > 65%

Figure 2 outlines the ‘pathways’ in studies from recruitment of participants, screening with risk assessment (RA) tools, undertaking stool tests (i.e. FIT/ iFOBT, FOBT/gFOBT or RPHA-FOBT) through to colonoscopy or sigmoidoscopy and Figs. 3 & 4 highlight the pathway of studies that achieved a stool test uptake of > 65% (*n* = 13) in more detail. Six studies achieved a stool test uptake of < 45%, three studies reported a stool test uptake between 45 and 65% and two studies either screened with colonoscopy only or conducted a RA and screened those at high-risk for CRC with a colonoscopy.

### Recruitment

Participants were recruited from community settings (*n* = 17, i.e. own home, public places and community clubs), clinics or hospitals (opportunistically when they visited for reasons other than screening; *n* = 5), a workplace (n = 1) [18] and one study compared three different recruitment strategies [19]. FOBT/FIT uptake ranged between 14 and 100% for interventions that recruited from the community/home with 9/18 interventions achieving > 65% uptake.

Eligibility criteria in most studies included females and males aged ≥40 or ≥ 50 years (*n* = 4 and *n* = 16, respectively), living in the study area or registered with a clinic. One study included adults aged ≥30 years [20] and one study included males only [21]. Population-based studies (that recruited from a specific geographical area within a country, *n* = 10) generally included all residents within the given age-range and specified no exclusion criteria (except for people with a CRC history). The remaining studies excluded participants who reported CRC symptoms, a family history of CRC and/or chronic bowel conditions (e.g. inflammatory bowel disease). Recruitment was conducted by health care professionals, community health volunteers/ navigators/workers (CHW) or researchers, either face-to-face, through a letter, text message and/or a telephone call. Three studies utilised media only for recruitment, one study compared media only vs. other modes of recruitment [19] and four studies complemented media with other recruitment strategies. Studies that recruited participants through media only voluntary response sampling achieved a stool test uptake of > 65% [22, 23] compared to a study that recruited participants through media only population-based sampling which achieved a stool test uptake of 14% [24]. Some studies specified only who delivered the intervention and it was unclear who recruited participants. Opportunistic interventions (5/6) that were conducted from clinics or hospitals achieved an uptake of > 65% [19, 21, 25–27]. Most population-based sampling studies achieved better stool test uptake proportions when participants were approached face-to-face (40, 53, 63, 76, 83 and 98%) compared to recruitment through letter, phone, or media call (14, 35 and 63%). Two population-based studies with the lowest participation rates and two population-based studies with the highest participation rates required participants to return two FITs rather than one FIT (14 and 40% vs 76 and 98%, respectively) [24, 28–30]. Interventions that recruited from places other than community/homes or clinics/hospitals, such as workplaces [18] or leisure clubs [31], reported uptake proportions of only 16.5 and 26%.

### Intervention (education/screening)

Most studies (8/9) in China included a RA as part of the primary screening process usually in the form of a high-risk factor questionnaire (HRFQ) and one study utilised the Asian-Pacific Colorectal Screening (APCS) scoring system [26]. Including a RA did not appear to make a difference in terms of uptake. Thirteen studies offered FIT as the primary screening test, seven offered FOBT, one offered RA with colonoscopy follow-up, one offered colonoscopy and two suggested participants to attend screening but the screening was not part of the study. Screening uptake did not differ according to type of stool test (FIT or FOBT). However, interventions that utilised colonoscopy as the primary screening test [18] or followed-up a positive HRFQ with a colonoscopy [32] had the lowest response rates (16.5 and 14%, respectively).

Individual or group education was part of the screening intervention in six studies [21, 31, 33–36] though 2/6 studies focused only on education and did not offer screening - participants had to take a stool test independently [31, 35]. Visual information about stool collection (e.g. a video or brochures/pamphlets) was provided to participants in 6/24 studies [23, 30, 36–38] and 5/24 studies provided educational material but the content was not described. Findings from studies providing education/ educational materials were mixed. Interventions that provided an educational component only and suggested that participants should complete an FOBT but did not offer a stool test as part of the intervention reported much lower FIT/FOBT completion rates (35.7 and 26.0%) [31, 35] compared to interventions that offered an educational component and a stool test (in 3/4 interventions) (i.e. 80–100% [34], 74% [21] and 96% [36]).

Screening was ‘free of charge’ for participants in the reports of 16 studies, seven studies did not describe how screening costs were covered and one study reported that cost of screening was paid by participants [31]. Participants in 7/24 studies were reminded (by phone call, email or letter) to return their stool sample if it was not returned within the specified timeframe. Screening participation ranged between 14 and 97.7% for interventions that used reminders, hence, the extent to which receiving a reminder made a difference to stool test uptake was unclear.

There was very limited evidence for the activity of tailoring or personalisation of interventions (**Supplementary Table** **3**). The involvement of participants in co-creating information materials in plain, local language was noted in only a few studies [34, 36, 39]. Other ‘personal’ methods included collecting stool samples from homes (rather than asking participants to return stool samples to a clinic) [28, 29], sending personally named and addressed letters [18] and asking clinicians who were known to the target population to issue invitations to participate in the intervention [38]. The intervention in three studies was tailored according to a theory - the Health Belief Model [21, 35], social cognitive theory [21] and the preventive health model [31]. One study reported an educational intervention (in Iran) that counselled individuals to address personal barriers to screening, included family members in health education sessions, sent reminders for stool completion and asked physicians of the same sex to consult and encourage participants [21]. Modifications conducted while the intervention took place were not reported. Authors did not report any modifications to the intervention that was used in their study. Any modifications that were described, referred to modifications regarding the analysis/data that was used in a study, an extension of the study location [40], and modifications to the RA tool [28], screening test [34] or measurement tools [35] prior to the implementation of an intervention.

### Implementation considerations

Data extraction relating to ten implementation measures [16] is summarised in **Supplementary Table** **5**. We used data relating to screening participation to refer to ‘***reach/uptake’*** and as an indicator of an intervention’s ‘***acceptability***’ (Table 1). Intervention acceptability per se was reported only if studies provided data that referred specifically to other factors associated with acceptability, e.g. participant satisfaction [33, 38], drop-out rates for stool collection and colonoscopies [22, 23, 26, 38, 40] and follow-up with treatment recommendation [34]. In one study, a much higher acceptance rate was achieved when house visits were used to increase reach/uptake (86.6%), compared to opportunistic screening in clinics (44.8%) or through an awareness campaign (13.8%) [19].

Regarding reported socio-demographic characteristics of participants, intervention participation appeared to be higher amongst females (42.5–74%) compared to males (26–57.5%) in 14/16 studies that reported uptake by sex and were not limited by sex. For example, between 70 and 75% of participants in two studies in Mexico were females [18, 23], and screening participation was higher among Iranian females (compared to males) after they received two different educational interventions [31, 36]. One study (in Romania) reported higher uptake amongst males compared to females (57.5% vs 42.5%) [22]. Two interventions (in China) reported higher screening participation amongst older age groups [24, 41].

Implementation measures other than ‘reach’ were not reported or reported only vaguely. ‘***Adoption***’ was reported if an intervention was adopted from a previous intervention or adaptations were made to meet the needs of the target population. Many studies described the first time when an existing intervention was piloted in a country. Four studies based in China, Bulgaria and Serbia [27, 29, 38, 40, 42, 43] reported interventions that followed (revised) screening guidelines/recommendations and 3/4 studies reported an uptake > 65% and one reported an uptake between 45 and 65%. Studies also reported that interventions were guided by experts [24, 27, 36]. Interventions that were guided by a theory were described earlier. One educational intervention described that the room where the education was delivered was well lit and chairs were comfortable [35].

Intervention ‘**appropriateness**’ was assessed in terms of its suitability for the target population and variation in reach between, for example, age groups [38] as well as rural vs urban areas, geographical locations [25, 40], education levels [38], participants at risk for CRC [32] or ethnic groups in studies conducted in Malaysia [19, 25, 44]. Higher uptake among rural communities vs urban was reported for stool tests [41, 45] and colonoscopy [30]. For example, a large-scale community intervention in Thailand trained community health workers to recruit participants. Participation was highest in rural compared to urban districts (73.2% vs 45.1%) and amongst people aged 60–65 years vs 50–54 years (78.9% vs 52.9%), which was due, probably, to a difference in recruitment approach – i.e. participants in rural areas were recruited face-to-face and in urban areas mainly through poster advertisements [39]. A few participants, particularly people aged ≥70 years, in one study found it difficult to provide a stool sample and handle the collection tube (Mann-Whitney U test = 12.3, *p* < 0.001) [38].

‘***Feasibility***’ refers to the *extent to which an intervention can be delivered in a given setting,* including demand on a provider system and the availability of supporting administrative data. Often, intervention descriptions included use of databases (e.g. cancer registries) to record and link participant data instead of the demand on a system. Studies in China [20, 28–30, 32, 46] and Thailand [39] linked interventions into data collected from cancer registries and studies in Bulgaria, Iran and Thailand linked interventions into, and used, data collected from health care registries or insurance companies [36, 40]. Interventions with 45–65% and > 65% stool test uptake were more likely to report linkage to a cancer/ health care/ insurance registry (3/3 and 4/14 studies, respectively) vs those with < 45% uptake (1/6).

***‘Fidelity’*** or the extent to which an intervention was implemented as intended was absent or lacking in most studies. Most studies reported neither a targeted sample size nor deviations from the study protocol. One population-based study in China reported recruiting 29% of the target population compared to the goal of 42% [41] and a population-based study in Serbia recruited 19% instead of the planned 75% of the target population [40]. Participants from an educational intervention in China attended an average of 3.25 lectures in the first year and 2.71 lecture in the second year, however, the study did not report how many lectures were delivered [33]. Another study in Serbia reported that colonoscopies were delivered within 37 days of referral (which was close to the 31 days according to European guidelines 2008) [27].

***Intervention costs*** related to CRC screening, treatment, marketing and transport were reported in only two studies, both in China [42, 43, 46]. Some studies reported that training was provided for staff who delivered interventions and quality control/ assurance was conducted. Studies that reported quality control achieved lower stool test uptake compared to those that didn’t (i.e. < 45% [24, 28, 41]; 45–65% [45]). One study reported that the awareness campaign was the cheapest recruitment approach compared to opportunistic recruitment in clinics or house-to-house recruitment [19].

‘***Intervention complexity***’ and ‘***sustainability***’ - most studies contained sparse reports about these indicators of implementation. Interventions tended to be rated as complex because they comprised several steps (to recruit and screen participants) or the running and roll out of the intervention was complex. ‘Sustainability’ was not addressed in studies, though interventions that were funded and run by the government (often as part of a national screening programme) seemed likely to have better long-term sustainability than NGO-, hospital- and research-led interventions that had no or limited government support. Furthermore, interventions that were delivered in clinics seemed to be easier to implement and more sustainable than one-off educational interventions or (non-clinic) community interventions because community volunteers required training and resources.

‘***Penetration***’ referred to the joint or shared organisation and delivery (e.g. between government bodies and NGOs) of CRC screening interventions in a target community as well as the number of sites reached. The interventions in 10/24 studies invited everyone who lived in the target area and met inclusion criteria and 3/9 studies reported FOBT/FIT uptake of over 65% and 3/9 reported FOBT/FIT uptake between 45 and 65%. Population-based studies that were less successful (14% uptake of colonoscopy) conducted a HRFQ (instead of FIT/FOBT) and offered a colonoscopy appointment if the HRFQ was positive [32]; or were asked to complete two FIT tests instead of one (39.7 and 14.0%) [24, 28] or recruited participants through a letter rather than face-to-face or over the phone (35.2% uptake) [41]. The 14/24 studies that were not population-based appeared to have had lower penetration in the target population and fewer collaborations. A population-based study (in Thailand) that involved a collaboration between local and international institutes reported a FIT uptake of 62.9% [39]. The screening intervention in a population-based study (in Serbia) was delivered via a collaboration between several institutions and reported a similar FIT uptake proportion of 62.5% [40]. A FIT uptake of between 80 and 100% over 5 years for a convenience (and potentially self-selected) sample was reported for a Malaysian study that involved a collaboration between NGOs, medical and paramedical organisations and community leaders [34].

### Reports and commentaries

Sixteen reports and commentaries referred to challenges and opportunities for implementing CRC screening in LMICs. Three commentaries discussed CRC screening in a global context [47–49], two in Africa (Kenya and Sub-Saharan Africa), one in China [50], three in central and South-America [51–53], three in Europe [54, 55] and four in the Middle East [56–59]. Table 2 summarises the key opportunities and challenges for CRC screening in different LMIC settings.

**Table 2:** Top 10 Challenges and opportunities for the implementation of CRC screening programmes in LMICs

Synthesised opportunities and challenges	Further explanation
***Challenges***	
Lack of (cancer registry) data, poor reporting of CRC data [67–71]	Cancer registries have not been established in many LMICs and reporting of cancer-related information is often not mandated. Reliable data on CRC incidence, mortality and screening is therefore often lacking. This leads to and underrepresentation of the cancer problem in LMICs and as a result, lack of funding.
Low level of CRC knowledge (general population) [67, 69, 72–74]	The general public lacks awareness about CRC, CRC screening and the importance of early detection of CRC.
Inadequate (i) number of trained staff and (ii) staff training [72, 73, 75–77]	Lack of specialised staff (e.g. endoscopists, oncologists, radiotherapists, gastroenterologists) and lack of specialised training opportunities leading to lack of appointments for screening and treatment.
Poor health care system infrastructure [67, 68, 77, 78]	Screening services are not widely available and there are long-waiting times for colonoscopies and endoscopies. There is a lack of screening equipment and structural deficiencies including screening centres. It can also be difficult to travel to services for patients who live in rural areas.
Lack of organised screening and absence of screening guidelines or poor uptake and use of guidelines [68, 72, 75, 79]	Lack of organised screening programmes/screening guidelines. Some regions completely lack access to CRC screening at primary care level.
Health policy agenda - CRC not prioritised [72, 73, 75, 79, 80]	Other health services are prioritised over CRC screening in countries where incidence is low. The relatively low importance ascribed to CRC is due partly to an underestimation of the problem of CRC (due to lack of data) as well as other, often communicable, conditions taking priority.
Low level of CRC knowledge and procedures among medical staff [68, 72, 74, 78, 80]	Low level of awareness among physicians about CRC and poor implementation of screening guidelines.
Inadequate financial resources [67, 73, 74, 80, 81]	Lack of funding to improve infrastructure and access to screening programmes, staff, centres, treatment, etc.
Cost to patients [68, 69, 74, 78]	Cost can be a barrier where screening and cancer treatment expenses need to be covered by patients (challenge to make CRC screening widely accessible)
Insufficient public health campaigns [68, 73, 79, 80]	Lack of CRC awareness raising activities and information about CRC in general likely contributes to low public awareness.
***Opportunities***	
Improve reporting of CRC screening efforts and evaluation [67, 72, 73]	Establish timely, reliable and efficient health information system for the design, management and evaluation of CRC prevention activities. Implement electronic medical records to allow for ICD-10 coding. Set up a cancer registry where there is none.
Cost-effective CRC screening methods [67, 74, 80, 81]	Identify cost-effective, culturally-acceptable CRC screening methods and conduct cost-effectiveness evaluation of services to understand impact of services and improve existing practice.
Improve health care infrastructure [67, 69, 76, 80]	Improve and align infrastructure, improve equitable distribution of screening technology throughout regions
Increase number of trained endoscopists and provide specialised training to health care staff [67, 70, 80]	Train specialised staff to conduct screening. Options are to train individuals from other specialities and non-physicians to deliver services and to provide e-training. Improved /annual standardised training should also be delivered for personnel who are already practicing.
Prioritise screening for high risk population [68, 74, 81]	Improve collection of family history and other information related to high-risk of CRC. Screen population at high-risk to better utilise resources and improve awareness on screening guidelines by family history/ high-risk.
Commitment from governments [67, 69, 70]	Committed, coordinated and comprehensive approach to make CRC a public health priority. One option is bulk purchasing of screening tests from governments so that procedures can be streamlined, costs reduced and efficiency increased
Awareness programmes for the public and HCPs [74, 78, 80]	Improve CRC awareness among HCPs and patients through for example CRC awareness campaigns/ programmes
Improve planning of CRC screening programmes, guidelines and policies [69, 73, 74]	The increasing CRC incidence is demanding better programmes. Establish national screening programmes, guidelines for CRC screening/ organized screening strategy and establish cancer control planning through dedicated agencies/ NGOs and/or government.
Patient navigation and communication with HCPs to improve adherence to screening programmes [70, 76, 80, 81]	Utilize patient navigation; review positive result letter to improve colonoscopy compliance; improve communication about CRC risk and the importance of early screening and follow-up screening/ treatment (colonoscopy) to improve compliance rates
Improve quality assurance of screening services [73, 76]	Improve programme quality control, quality assurance to ensure optimal impact and improve the quality of health care services

CRC – colorectal cancer, HCPs – health care professionals, LMIC – low-and middle-income country

## Discussion

This review found that a range of different strategies was employed to increase CRC screening and that implementation of CRC screening was reported in only nine LMICs. The results indicated also that there were a number of opportunities and challenges in relation to the structural implementation of screening within health care settings. Although much of the evidence from empirical studies comes from Asia, in particular China, the findings from the mapping exercise suggested that the lessons might be generalised to other upper-middle income countries. Empirical studies were absent in low-income countries and there is an urgent need to address this evidence gap. However, reflections emanating from the policy reviews/commentaries suggested, perhaps unsurprisingly, that outer setting constructs [60] (e.g. infrastructure, financial resources & trained professionals, data collection), potentially, may be more prominent barriers in low-income countries than in upper-middle income countries that have more resources and better infrastructure. Similarly and understandably, findings from the commentaries/policy reviews suggested also that governments, across LMICs, did not tend to prioritise early detection of CRC and, instead, concentrated resources on treatment services and on treating higher prevalent communicable and non-communicable diseases.

The review found that the most common screening approach mirrored the approach that was used in HICs whereby participants were asked to collect and return one stool sample for a FOBT or FIT (that was followed-up with a colonoscopy if positive) except that none of the studies asked participants to mail stools samples – a feature which has been implemented in HICs and found to be cost-effective [61]. Participation rates did not differ by stool test type (FIT/FOBT) in contrast to European Union member states where studies that utilised FIT reported higher participation [62]. This difference might be related to the fact that the stool collection process was the same in LMICs (irrespective of the type of test – FIT or FOBT) unlike HICs. Only studies that were conducted in Asia employed RA tools as part of the screening process. For example, the Asia-Pacific Colorectal Screening (APCS) score [26] identified people who had a 3.4-fold (95% CI 1.8–6.4) increased risk of advanced colorectal neoplasia in a prospective multi-centre study in China [63]. RA tools appear to be an efficient and cost-effective way to identify individuals at high risk of CRC [64] and the evidence indicates that, overall, they merit consideration by other LMICs. Furthermore, it was unclear whether sending reminders made a difference to screening participation, which has previously been suggested to be an effective population-based intervention strategy for increasing CRC screening uptake [12].

In terms of intervention recruitment, FIT/FOBT interventions that had face-to-face interactions with participants achieved, on average, a better screening uptake compared to other modes of interaction. In particular, 5/6 opportunistic screening interventions in clinics achieved an uptake of > 65%. This is high compared to the uptake of interventions in HICs where FOBT screening uptake was less than 50% [11]. However, it is important to note that most intervention studies in the review employed a quasi-experimental design and a variety of recruitment strategies. Recruitment of participants through voluntary response or convenience sampling, for example, are likely sources of bias and the results of these studies need to be interpreted with caution – e.g. participants with higher awareness about CRC are more likely to participate in screening which translates into higher participation rates.

The included studies paid only limited attention to implementation considerations, especially regarding complex population-based screening interventions. There was an absence of detail regarding every core implementation construct listed by Tierney et al. [16] and, clearly, there is a need to improve reporting practice in journal papers and other forums in order to disseminate learning about how to implement programmes. Tierney et al. argued that a service or intervention that focused on system-level factors (e.g. complexity, cost, impact on workflow, appropriateness and sustainability) would increase the likelihood of achieving successful programme implementation and sustainability [16]. However, it is difficult to test these arguments when there is very little transparency in the reporting of implementation measures. It is noted that the absence of data or reports about implementation does not mean, necessarily, that these issues were not considered or addressed in practice. Intervention reach/uptake was the main implementation construct that was reported in the studies. Participation was influenced mostly by health system factors, and the biggest barriers to improving uptake were low knowledge level and poor education of clients and providers in keeping with an umbrella review by Priaulx et al. [9]. It is surprising that the reasons for non-participation were not assessed or reported in any of the studies in the review. Possibly, non-adherence is seen as a ‘first world problem’ in countries that struggle to provide CRC screening. Identifying non-adherers and addressing ‘hard-to-reach’ factors is likely to improve health equity. A recent systematic review found that the common barriers to uptake of CRC screening in HICs were related to some of the implementation considerations mentioned earlier, i.e. logistical barriers and lack of awareness about CRC screening [65].

The review mapping activity indicated that reported inhibitors of implementation tended to be interlinked. For example, a lack of financial resources has a knock-on effect on implementation constructs such as the outer setting (e.g. infrastructure, prioritising funding for other common conditions), the inner setting (e.g. lack of trained staff) and individuals involved (e.g. lack of public health campaigns and communication with doctors resulting in low awareness). An organised screening programme will increase the number of FITs/FOBTs and, in turn, increase the number of colonoscopies/ sigmoidoscopies. Thus, testing facilities and appropriate treatment need to be in place before efforts are directed towards the goal of improving screening uptake. Whole-system planning is required including better monitoring and reporting of screening activities as well as continuous quality assurance [62]. Frameworks such as the Implementation Research Logic Model should guide future implementation studies to improve the rigor of designing and describing complex health service delivery interventions [66]. Some barriers to implementation do not require additional financial resources to overcome their inhibiting effects. There appeared from the results of the review to be scope for improving reporting and follow-up of CRC positive cases, improved risk communication, as well as community support and navigation [39]. Finally, Mindful of the current pandemic and the pressures that it places on health systems and human behaviour, there is a need to find creative ways in which to address the key finding that approaching individuals face-to-face (either alone or in combination with other recruitment tools), in a community or clinic setting was the most effective method of engaging individuals to participate in CRC screening in upper-middle income countries.

There are some limitations to this review. We did not conduct a formal quality assessment of the included research (in keeping with scoping review methodology [13]). Furthermore, the search was conducted at the end of January 2020 and was not updated due to resource constraints. It is possible evidence may have been missed by limiting the search to English language articles.

## Conclusion

Findings from this scoping review indicate that particular interventions applied to small-scale, as well as population-based CRC screening programmes, in middle-income countries achieve a FOBT/FIT uptake of > 65%. Uptake intervention implementation needs to take account of, and be responsive to, differences in health-care systems, economy and infrastructure of countries. The review identified also the commonly reported challenges and opportunities that LMICs need to consider when planning and implementing CRC screening availability and improving uptake.

## Supplementary Information

**Additional file 1 Supplementary Table 1.** Preferred Reporting Items for Systematic reviews and Meta-Analyses extension for Scoping Reviews (PRISMA-ScR) Checklist

**Additional file 2 Supplementary Table 2.** Definition of income groupings

**Additional file 3 Supplementary Table 3.** Search terms and strategy as devised for MEDLINE, Embase and Web of Science

**Additional file 4 Supplementary Table 4.** TIDieR Checklist for included studies

**Additional file 5 Supplementary Table 5.** Data extraction implementation measures

## Data Availability

All data generated or analyzed during this study are included in this published article and its supplementary information files.

## Abbreviations

APCS: Asian Pacific Colorectal Screening
C: Community
CG: Control group
CHC: Community health clinic
CHW: Community health worker
CRC: Colorectal Cancer
FIT (iFOBT): Faecal Immunochemical Test
FOBT (gFOBT): Faecal Occult Blood Test
GP: General practitioner
H: Hospital
HN: Health navigator
HW: Health worker
HCP: Health care professional
HIC: High-income country
HRFQ: High risk factor questionnaire
LMIC: Low- and middle-income country
IG: Intervention group
N: Number
NGO: Non-government organization
NR: Not reported
PRISMA-ScR: Preferred Reporting Items for Systematic Reviews and Meta-Analysis: extension for scoping reviews
RA: Risk assessment
Y: Years
