# Supplementary material for: The implementation of colorectal cancer screening interventions in low-and middle-income countries: a scoping review
Source: BMC Cancer. 2021 Oct 19;21:1125. doi: 10.1186/s12885-021-08809-1 (PMC8524916; doi:10.1186/s12885-021-08809-1)
Supplement: Supplementary file 2 — Additional file 2 Supplementary Table 2. Definition of income groupings [file 12885_2021_8809_MOESM2_ESM.docx]

**Supplementary table 2. Definition of income groupings**

| Definition of income groupings has been sourced from the World Bank and is based on gross national income (GNI): <https://datahelpdesk.worldbank.org/knowledgebase/articles/906519-world-bank-country-and-lending-groups>  ***Low-income:*** Low-income economies are defined as those with a GNI per capita, calculated using the World Bank Atlas method, of $1,025 or less in 2018.  ***Lower-middle income:*** Lower-middle income economies are defined as those with a GNI per capita, calculated using the World Bank Atlas method, between $1,026 and $3,995.  ***Upper-middle income:*** Upper-middle income economies are defined as those with a GNI per capita, calculated using the World Bank Atlas method, between $3,996 and $12,375.  ***High-income***: High-income economies are defined as those with a GNI per capita, calculated using the World Bank Atlas method, of $12,376 or more. |
| --- |
