# Supplementary material for: The implementation of colorectal cancer screening interventions in low-and middle-income countries: a scoping review
Source: BMC Cancer. 2021 Oct 19;21:1125. doi: 10.1186/s12885-021-08809-1 (PMC8524916; doi:10.1186/s12885-021-08809-1)
Supplement: Supplementary file 3 — Additional file 3 Supplementary Table 3. Search terms and strategy as devised for MEDLINE, Embase and Web of Science [file 12885_2021_8809_MOESM3_ESM.docx]

**Supplementary materials 3:** Search terms and strategy

**Database: MEDLINE | Platform: OVID | 27/01/2020**

| 1 | exp Colorectal Neoplasms/ |
| --- | --- |
| 2 | bowel cancer.mp. |
| 3 | early diagnosis/ OR "early detection of cancer"/ |
| 4 | Mass Screening/ |
| 5 | (f$cal occult blood test OR fobt OR f$cal immunochemical test OR fit OR colonoscopy OR sigmoidoscopy).mp. [mp=title, abstract, original title, name of substance word, subject heading word, floating sub-heading word, keyword heading word, organism supplementary concept word, protocol supplementary concept word, rare disease supplementary concept word, unique identifier, synonyms] |
| 6 | Developing Countries/ |
| 7 | (Angola* OR India* OR Papua New Guinea OR Bangladesh* OR Indonesia* OR Philippin* OR Bhutan OR Kenya* OR Sao Tom* OR Bolivia* OR Kiribati OR Senegal* OR Cabo Verde* OR Kyrgyz* OR Solomon Island* OR Cambodi* OR Lao* OR Sudan* OR Cameroon* OR Lesotho OR Timor Leste* OR Comoros OR Mauritania* OR Tunisia* OR Congo OR Micronesia OR Ukrain* OR Cote d'Ivoire OR Moldov* OR Uzbekistan* OR Djibouti OR Mongolia* OR Vanuatu Egypt* OR Morocc* OR Vietnam* OR El Salvador OR Myanmar* OR Gaza* OR Eswatini OR Nicaragua* OR Zambia* OR Ghana* OR Nigeria* OR Zimbabwe* OR Honduras* OR Pakistan* OR Argentin* OR Paraguay* OR Mexic* OR Venezuel* OR Costa Rica* OR Cuba* OR Jamaica* OR Guatemala* OR Peru* OR Dominican Republic* OR Ecuador* OR Colombia* OR Brazil* OR Chin* OR Sri Lanka* OR Malay* OR Thai* OR Turk* OR Iran* OR Albania* OR South Africa* OR Maurit* OR Namib* OR Algeria* OR Botswana* OR Bulgaria* OR Fiji* OR Gabon* OR Nauru* OR American Samoa* OR Georgia* OR North Macedonia* OR Grenada* OR Armenia* OR Azerbaijan* OR Guyana* OR Romania* OR Belarus* OR Russia* OR Belize* OR Iraq* OR Samoa* OR Bosnia* OR Herzegovina* OR Serbia* OR Jordan* OR Kazakhstan* OR Kosovo* OR St Lucia* OR Lebanon* OR Grenadin* OR Suriname OR Maldives OR Tonga* OR Dominica OR Marshall Islands OR Turkmenistan OR Equatorial Guinea OR Tuvalu OR Montenegro* OR Liby* OR Afghan* OR Guinea* OR Sierra Leone OR Benin* OR Haiti* OR Somalia* OR Burkina Faso* OR Korea OR South Sudan* OR Burundi* OR Liberia* OR Syria* OR Central African Republic OR Madagascar* OR Tajikistan* OR Chad* OR Malawi* OR Tanzania* OR Congo* OR Mali* OR Togo* OR Eritrea* OR Mozambique* OR Uganda* OR Ethiopia* OR Nepal OR Yemen* OR Gambia* OR Guinea* OR Rwanda*).mp. [mp=title, abstract, original title, name of substance word, subject heading word, floating sub-heading word, keyword heading word, organism supplementary concept word, protocol supplementary concept word, rare disease supplementary concept word, unique identifier, synonyms] |
| 8 | 1 OR 2 |
| 9 | 3 OR 4 OR 5 |
| 10 | 6 OR 7 |
| 11 | 8 and 9 and 10 |
| 12 | limit 11 to (English language and humans) |

**Database: Embase | Platform: OVID | 27/01/2020**

| 1. exp Colorectal Neoplasms/ |
| --- |
| 2. bowel cancer.mp. |
| 3. early diagnosis/ or "early detection of cancer"/ |
| 4. Mass Screening/ |
| 5. (f$cal occult blood test or fobt or f$cal immunochemical test or fit or colonoscopy or sigmoidoscopy).mp. [mp=title, abstract, heading word, drug trade name, original title, device manufacturer, drug manufacturer, device trade name, keyword, floating subheading word, candidate term word] |
| 6. Developing Countries/ |
| 7. (Angola* or India* or Papua New Guinea or Bangladesh* or Indonesia* or Philippin* or Bhutan or Kenya* or Sao Tom* or Bolivia* or Kiribati or Senegal* or Cabo Verde* or Kyrgyz* or Solomon Island* or Cambodi* or Lao* or Sudan* or Cameroon* or Lesotho or Timor Leste* or Comoros or Mauritania* or Tunisia* or Congo or Micronesia or Ukrain* or Cote d'Ivoire or Moldov* or Uzbekistan* or Djibouti or Mongolia* or Vanuatu Egypt* or Morocc* or Vietnam* or El Salvador or Myanmar* or Gaza* or Eswatini or Nicaragua* or Zambia* or Ghana* or Nigeria* or Zimbabwe* or Honduras* or Pakistan* or Argentin* or Paraguay* or Mexic* or Venezuel* or Costa Rica* or Cuba* or Jamaica* or Guatemala* or Peru* or Dominican Republic* or Ecuador* or Colombia* or Brazil* or Chin* or Sri Lanka* or Malay* or Thai* or Turk* or Iran* or Albania* or South Africa* or Maurit* or Namib* or Algeria* or Botswana* or Bulgaria* or Fiji* or Gabon* or Nauru* or American Samoa* or Georgia* or North Macedonia* or Grenada* or Armenia* or Azerbaijan* or Guyana* or Romania* or Belarus* or Russia* or Belize* or Iraq* or Samoa* or Bosnia* or Herzegovina* or Serbia* or Jordan* or Kazakhstan* or Kosovo* or St Lucia* or Lebanon* or Grenadin* or Suriname or Maldives or Tonga* or Dominica or Marshall Islands or Turkmenistan or Equatorial Guinea or Tuvalu or Montenegro* or Liby* or Afghan* or Guinea* or Sierra Leone or Benin* or Haiti* or Somalia* or Burkina Faso* or Korea or South Sudan* or Burundi* or Liberia* or Syria* or Central African Republic or Madagascar* or Tajikistan* or Chad* or Malawi* or Tanzania* or Congo* or Mali* or Togo* or Eritrea* or Mozambique* or Uganda* or Ethiopia* or Nepal or Yemen* or Gambia* or Guinea* or Rwanda*).mp. [mp=title, abstract, heading word, drug trade name, original title, device manufacturer, drug manufacturer, device trade name, keyword, floating subheading word, candidate term word] |
| 8. 1 or 2 |
| 9. 3 or 4 or 5 |
| 10. 6 or 7 |
| 11. 8 and 9 and 10 |
| 12. limit 11 to (english language and humans) |

**Database: Web of Science | Platform: Clarivate | 27/01/2020**

((ALL=(Colorectal cancer OR bowel cancer)) AND ALL=(early detection OR f$cal occult blood test OR fobt OR f$cal immunochemical test OR fit OR colonoscopy)) AND ALL=(Developing countr* OR low income countr* OR middle income countr*)
