# Supplementary material for: The implementation of colorectal cancer screening interventions in low-and middle-income countries: a scoping review
Source: BMC Cancer. 2021 Oct 19;21:1125. doi: 10.1186/s12885-021-08809-1 (PMC8524916; doi:10.1186/s12885-021-08809-1)
Supplement: Supplementary file 4 — Additional file 4 Supplementary Table 4. TIDieR Checklist for included studies [file 12885_2021_8809_MOESM4_ESM.docx]

**Supplementary table 4.** TIDieR Checklist for included studies

| *Reference information*  **Location** | **Brief name** | **Why**  (Rational) | **CRC Test** | **What / How much**  (Study design, materials, procedures) | **Who recruited/ delivered** | **How**  (mode of delivery) | **Where** | **When** | **For whom** | **Tailoring** | **Modification** |
| --- | --- | --- | --- | --- | --- | --- | --- | --- | --- | --- | --- |
| **Stool test uptake >65%** | | | | | | | | | | | |
| *Cai et al 2011*  *Ma et al 2012*  *Cai et al 2016*  **China**  (3 rural communities in Jiashan) | Population-based opportunistic screening in community | CRC mortality in China is increasing.  Screening high-risk individuals is likely more efficient. | 2 x FIT,  1 x HRFQ  Colonoscopy if positive  FOBT  Colonoscopy if FOBT positive | **Design:** Cross-sectional  **a.** Trained physicians went to community and completed HRFQ  **b.** CHW collected 2 stool samples at interval of one week for FIT  **c.** If FIT OR HRFQ were positive, colonoscopy was recommended  *Test free of charge for participants* | **Recruited:** unclear  **Delivered:**  Physicians  CHW | Face-to-face | Community | 2007- 2009 | Residents aged 40-74 y.  *(medically and eco-nomically underserved population)* | Community visits at convenient times for participants | Study is a revised screening programme accepted by the National Cancer Screening Programme in 2006. |
| *Gong et al 2018*  **China** (Shanghai, 17 districts) | Population-based opportunistic screening in community | Increasing CRC incidence and mortality rates in China and low CRC screening uptake. | 2 x FIT,  1 HRFQ  Colonoscopy if positive | **Design:** Cross-sectional  **a.** Promotion through mass media (radio, TV, posters, health information brochures)  **b.** CHCs mobilized target population with collaboration of neighbourhood committees  **c.** HRFQ assessment; individuals were given 2 stool containers to collect sample at home, within an interval of 7 days, and asked to return each sample to a CHC within 48 hours after collection (reminder call - twice if no sample returned within 2 weeks)  **d.** 60-second video to explain sample collection (played on repeat at CHC)  **e.** 1 or 2 positive FIT OR positive HRFQ were referred for colonoscopy  **f.** Contact via phone if missed follow-up  *Test free of charge for participants* | **Recruited:**  Media & CHC staff  **Delivered:** CHC staff | Mass media  Face-to-face | Community/ Community health clinics | Jan – Dec 2013 | Residents aged 50–74 y. | Collaboration with neighbourhood and village committees to improve uptake | Findings from 1 district was not included in final analysis as data was not submitted due to different information management system used. |
| *Zheng et al 2003*  **China**  (Jiashan county) | Mass screening programme | CRC survival chances are higher when detected early.  Develop and optimise a mass screening protocol and evaluates its efficacy in low-incidence areas. | 1 x HRFQ,  1 x reverse hemaggluti-nation FOBT (RPHA-FOBT)  Sigmoidoscopy if FOBT positive | **Design:** Cluster randomization trial  **a.** Field interviewers recruited participants and conducted home interviews  **b.** IG was asked to complete HRFQ and submit one-article-per-slide stool sample  **c.** Sigmoidoscopy for FOBT positive cases  *Cost for participants not described* | **Recruited & delivered:** Field interviewers | Face-to-face | Community | Data used from 1989-1996  Residents aged ≥ 30 y. | Residents aged ≥ 30 y. | Stool test specifically developed for population (RPHA-FOBT) | Not reported |
| *Hassan et al 2016*  **Malaysia**  (Kota Setar, Kuala Muda) | Opportunistic screening by clinics | Improve screening to detect CRC early and reduce mortality | FIT  (2^nd^ FIT if negative)  Colonoscopy if one iFBOT was positive | **Design:** Cross-sectional  **a.** Pre-test counselling & test explanation for potential participants at health clinics or hospital  **b.** Stool container & instructions on stool collection was given  **c.** Participant asked to return test kit immediately after stool collection  **d.** A second FIT was conducted if 1^st^ was negative  **e.** Participants were counselled by a medical practitioner and referred for colonoscopy if FIT positive  *Cost for participants not described* | **Recruited & delivered:** Medical practitioner  *(MOH initiative)* | Face-to-face | Clinics | 2013  . | Patients who underwent FIT in 2013 aged ≥50 y. | Not reported  (MOH protocol was followed) | Not reported |
| *Noriah et al 2010*  **Malaysia**  (Seremban) | Comparison of house-to-house intervention, awareness campaign and opportunistic testing in clinics | Early detection of CRC improves chances of cure. Aim was to determine feasibility, acceptability and cost implications of screening with FOBT. | 1 x FOBT  Colonoscopy if FOBT positive | **Design:** Cross-sectional  **Group 1: HOUSE-TO-HOUSE**  random sampling of participants – *(recruitment unclear)*  **Group 2: AWARENESS CAMPAIGN**  2000 pamphlets on colorectal cancer were distributed at supermarkets, bus stations & housing areas  **Group 3: OPPORTUNISTIC TESTING** **IN CLINICS**  Patients invited by the health care workers. Patients came to the health clinic for other treatments or for follow-up. Health education on colorectal cancer was given to the patients.  FOBT was completed as part of each strategy and participants were referred to colonoscopy if FOBT positive  *Test free of charge for participants* | **Recruited:**  Health care workers/ media  **Delivered:** Health care workers  *(Organised/ funded by government)* | Face-to-face / media | Community / clinics | 15^th^ Sept – 31^st^ Dec 2007 | Adults aged ≥50 y. | Different approaches were tested to identify which is most acceptable. | Not reported. |
| *Tze et al 2016*  **Malaysia**  (Low-income communities in Sentul, Selayang, Gombak, - PPR Intan Baiduri, PPR Taman Wahyu and PPR Taman Prima Selayang, Lembah Subang, Setapak, Cheras - Perumahan Awam Seri Sabah and PPR Hiliran Ampang. | Community education intervention | CRC screening is important for early detection. Low socio-economic groups are prone to late stage diagnosis and poorer survival rate in Malaysia. | 1 x FIT  Colonoscopy if FIT positive | **Design:** Cross-sectional  **a.** Engagement of community leaders, mass distribution of flyers, posters, banners  **b.** Trained volunteer medical students went door-to-door to promote campaign and recruit individuals  **c.** Screening workshop eligible participants (how to use, when and where to return FIT kit)  **d.** Drop-off kits within 2-3 days at collection stations within communities  **e.** Results were returned to participants within 2 weeks  **f.** Trained nurses visited CRC positive patients in their homes & scheduled appointment for colonoscopy  *Test free of charge for participants* | **Recruited & delivered:**  Volunteer medical students (with support from  community leaders)  *(Organised by an NGO)* | Face-to-face  Small media | Community | 2010-2015  (Annual 1-year long project, over 5 years, different district every year) | Residents aged ≥ 50 y. | Intervention conducted in 3 local languages. | Different type of test was used between 2010-2012 and 2013-2015 |
| *Aniwan et al 2017*  **Thailand**  Bangkok, Chiang Mai  Songkhla,  Khon Kaen | Clinical / health promotion intervention | Impact of different hemoglobin levels of the  FIT on advanced neoplasia, cancer detection, and the diagnostic miss rates between high-risk and average-risk  subjects has not been studied. | 1 x FIT,  1 x Colonoscopy | **Design:** Cross-sectional  **a.** Individuals who attended different health promotion programs at hospitals were recruited  **b.** Subjects were interviewed to assess their clinical risk using the APCS score by nurses  **c.** 1 x FIT followed by colonoscopy – subjects received explanation on stool collection and collected sample within 3 days before colonoscopy and analysed within 7 days  *Cost for participants not described* | **Recruited:** unclear  **Delivered:** Nurses | Face-to-face | Hospital | Dec 2014 – Dec 2016 | Participants from 6 hospitals across Thailand aged 50-75 y. | Local hospitals to recruit participants when visiting for other reasons– greater compliance through opportunistic screening  Use of Asia Pacific CRC Screening Score | Not reported |
| *Remes-Troche et al 2020*  **Mexico,** Veracruz | Mass media screening intervention | CRC incidence is increasing and insurance plans cover 85% of population for CRC screening and treatment. No organised national screening programme is in place. | 1 x FIT  Colonoscopy if FIT positive | **Design:** Cross-sectional  **a.** Recruitment through weekly newspaper adverts (for 3 months, in 2 newspapers)  **b.** Eligible participants were interviewed  **c.** Printed instructions for home sample collection were provided  **d.** Participants were asked to return FIT within 3 days after receiving test kit  **e.** Colonoscopy for FIT positive participants  *Test free of charge for participants* | **Recruited:** Media  **Delivered:** Unclear | Media  Face-to-face | Community | 15 May 2015 – 15 Jan 2016  Adverts for 3 months | Adults aged ≥50 y. | Not reported | Not reported  *(Authors noted that other recruitment strategies are required to reach various population groups and strategies tailored to national priorities and resources are needed)* |
| *Dimova et al 2015*  **Bulgaria** (Plovdiv) | Opportunistic self-testing intervention delivered by clinics | CRC incidence is high in Bulgaria but no population based screening is in place.  To test feasibility of testing with FIT | 1 x FIT  Extra 1 x FIT and fibrocolonoscopy if positive | **Design:** Cross-sectional  **a.** GPs contacted 20 health-insured patients via email or call  **b.** Participant agreed, visited GP & received test kit, educational brochure and questionnaire  **c.** Participants completed self-testing at home (following instructions) & returned completed questionnaire & kit to their GP within 2 weeks  **d.** Reminder call or email was sent after 2 weeks if no response was received from the participants  **e.** Positive FIT – participants were asked to repeat the test and referred with for fibrocolonoscopy  *Test free of charge for participants* | **Recruited and delivered:** GPs | Personal contact (call, e-mail, face-to-face) | Clinics | Jun – Sept 2013 | Health-insured, asymptomatic adults aged ≥45 y.  (each GP randomly selected 20 individuals) | Face-to-face contact and personal notification through GP  Evidence informed task force recommendations were employed for intervention use. | Not reported |
| *Sucevaeanu et al 2005*  **Romania**  (Dobrogea) | Media and clinical intervention | Increasing incidence and mortality of CRC in Romania. Few studies conducted in Easter Europe on CRC screening. Aim was to test feasibility of CRC screening intervention in Romania. | FOBT  (3 consecutive stool samples)  Colonoscopy or barium enema investigation if FOBT + | **Design:** Cross-sectional  **a.** Local newspaper was used to inform target population about screening  **b.** GPs informed all interested subjects about screening  **c.** GPs offered participants gFOBT for the collection of three consecutive stool samples; FOBT was classified as positive if at least one of the 3 samples was positive  **d**. Participants with 1 positive stool sample were referred to for colonoscopy or barium enema (if colonoscopy could not be performed)  *Cost for participants not described* | **Recruited:** Media  **Delivered:**  GPs | Media  Face-to-face | Hospital | May 2003 – Nov 2004 | Adults aged ≥50 y. | Colonoscopy / barium enema investigation was conducted as suitable | Not reported |
| *Scepanovic et al 2017*  **Serbia**  (Population-based) | Opportunistic screening with self-testing FIT organised by clinics | Increase in CRC incidence and mortality. Improve early detection and test acceptability of take-home FIT. | 1 x FIT  Colonoscopy if FIT + | **Design:** Cross-sectional pilot study  **a.** Participants recruited by GPs during clinic visits  **b.** Questionnaire completed to assess eligibility  **c.** FIT home-test kit (& instructions) given to participants  **d.** Return of sample within 7 days  **e.** Participants were contacted over the phone if result was negative and asked to consult their GP if test was positive who scheduled a colonoscopy  (follow up after 2 y. if negative)  *Test free of charge for participants* | **Recruited & delivered:** GP | Face-to-face | Primary health care centres | Aug – Nov 2013 | Adults aged 50 – 74 y. | Convenient for those who visited GP for other purposes | Not reported |
| *Gholampour et al 2018*  **Iran**  **(**Fasa City) | Educational intervention based on the Health Belief Model | CRC screening programme is in place with low participation rates. HBM- based education has shown to improve screening in other studies. | 1 x FOBT  Colonoscopy if FOBT positive | **Design:** Quasi-experimental study with control group  *Based on Health Belief Model and Social Cognitive Theory*  **a.** Participant recruited based on Household Health Files in health care centres were invited to completed a HBM-based questionnaire  **b.** Information about FOBT and stool collection container was given to participants (option to take stool sample at home or in laboratory)  **c.** IG received 8 x lectures (2 sessions per week & 2 x monthly follow-up sessions); counselling and face-to-face training about screening; follow-up meetings on the tests, sending recall cards, providing advice, encouragement, and help for screening. One session was attended by a family member as well as health centre officials and doctors for support.  **d.** FOBT was conducted for both IG and CG if stool containers were returned.  **e.** FOBT results delivered over phone (on request the results were sent in written test forms)  **f.** Referral to colonoscopy for FOBT positive cases  *Test free of charge for participants* | **Recruited & delivered:** Researchers | Face-to-face | Recruitment through clinics / training delivered in health centre halls | 2016-2017 | Males aged >50 y. | Very personalised intervention (see description)  Physician of same sex  Telegram group for exchange of information | Not reported |
| *Salimzadeh et al 2017*  **Iran**  (Tehran, rural & urban) | Integration of health navigation system into screening programme | Increase in CRC incidence and prevalence in Iran.  Patient navigation (PN) may help to reduce costs and improve access to health services in low-resource settings.  Aim to identify implications for scaling-up CRC screening at the national level. | 1 x FIT  Colonoscopy if FIT positive | **Design:** Cross-sectional pilot study  **a.** Eligible individuals were identified & contacted by HN over phone (rural) or public announcements (urban)  **b.** Participants attended in-person interview with HN at health centres; awareness was assessed; FIT testing barriers addressed and information on CRC symptoms, risk factors and screening were provided (30 min); worries were addressed  **c.** Participants received FIT kit & educational pamphlet on how to obtain stool  **d.** Participants were asked to return stool sample within 2 days after sampling to health houses (reminder call after one week if FIT kit was not returned)  **e.** 1 week after sample collection, FIT results were sent back to health centres. HN notified all participants with a negative FIT of results and recommended FIT screening in the next year; individuals with a positive FIT result were notified to schedule a colonoscopy within 4 weeks.  **f.** Colonoscopy: HN delivered detailed instructions on bowel preparation for colonoscopy.  *Test free of charge for participants* | **Recruited & delivered:**  Health navigators (HN) | Phone calls, public announcements  Face-to-face | Community health centres | 1 x 30 min session with HN  *Study timeframe not described* | Adults aged 45-75 y. | Native public health workers were hired and trained  CRC awareness was assessed and screening was explained in plain language.  Personal concerns addressed during interview  2 different recruitment approaches were rural/ urban | Not reported |
| **Stool test uptake 45-65%** | | | | | | | | | | | |
| *Khuhaprema et al 2014*  **Thailand** (Lampang province) | Pilot implementation program of CHW | No population based opportunistic CRC screening in Thailand. CRC is increasing. Piloting program to reduce deaths through primary prevention and screening. | 1 x FIT  Colonoscopy if FIT positive | **Design:** Cross-sectional  **a.** Recruitment in urban areas mainly through posters and in rural areas face-to-face  b. Pamphlets & stool collection pots distributed by CHW to eligible participants on household visits (*as part of routine visits that take once every 6 months*) **c.** Participants were given instructions on how to collect stool sample & asked to return sample within 3h of completion to Primary care unit or CHW **d.** FIT was carried out at the PCU by nurse or CH in front of the participant (participant was informed at visit of result) **e.** Colonoscopy appointment was fixed if FIT positive  *Cost for participants not described* | **Recruited & delivered:** CHW | Face-to-face | Community | April 2011- Nov 2012 | Residents aged 50-65 y. | Use of existing service (i.e. CHW visit every 6 months  Print materials in Thai language  CHW were trained to improve skills needed to deliver intervention | Not reported |
| *Bankovic Lazarevic et al 2016*  **Seriba**  (Population-based) | National organised CRC screening programme of Serbia | High CRC incidence and mortality rate and late stage diagnosis.  Reduce CRC incidence and mortality through early detection | 1 x FIT  Colonoscopy if FIT positive | **Design:** Cross-sectional  **a.** Target population (identified through database of those with health insurance and other citizens) invited via phone and letters to perform  **b.** FIT delivered by general practitioners in primary health care centres  **c.** Colonoscopies performed if FIT positive  *Cost for participants not described* | **Recruited & delivered:** Physicians  *(Programme conducted by MOH)* | Personal communication (letter/ phone call, face-to-face) | Primary health care centres | 2013-2014 (2 years) | Adults aged 50-74 y. | Not reported | Intervention was extended from 20 municipalities to 28 in the 2^nd^ year. |
| *Huang et al 2014*  **China** (Hangzhou, Shanghai, Harbin) | Opportunistic screening by invitation – comparing FIT vs FIT & high-risk score | FOBT alone may fail to detect lesions due to intermittent bleeding. Screening protocol should be based on cost-effectiveness in resource- limited settings. | 1 x FOBT vs. 1 x FOBT & HRFQ  Colonoscopy if positive | **Design:** Modelling study / cross-sectional  **a.** CDC officials contacted participants  **b.** Participants were asked to complete HRFQ and take FOBT  **c.** Participants with positive HRFQ or FOBT were referred for colonoscopy  *Cost for participants not described* | **Recruited & delivered:** CDC officials | Unclear (likely face-to-face) | Unclear (likely community) | July 2006 – Dec 2008 | Residents aged 40-74 y. | Study was conducted to identify one out of 8 most cost-effective scenarios to inform future interventions. | Not reported |
| **Stool test uptake <45%** | | | | | | | | | | | |
| *Wu et al 2019*  **China**  (Pudong New Area, Shanghai) | Community mobilisation intervention | Previous studies showed high sensitivity of screening procedures and high false positive rate. Aim was to optimize the risk assessment tool and seek an optimal initial  screening protocol for CRC | 2 x FIT,  1 x HRFQ  Colonoscopy if FIT or HRFQ positive | **Design:** Prospective cohort study  **a.** Recruitment through community mobilization  **b.** Risk assessment of eligible participants  **c.** Two stool samples were collected within 1 week by community health care staff from participants  **d.** Colonoscopy referral if FIT or HRFQ positive  *Test free of charge for participants* | **Recruited:** *[community mobilisation]*  **Delivered:**  Community health care staff | Face-to-face interview and stool collection | Community | 3 rounds of screening between 2013-2019  *(2 rounds, i.e. 2013-2017 included in analysis)* | Residents aged 50-79 y. | Stool collection by CHW to improve participation | Risk assessment tool was modified from previous studies to improve accuracy of tool. |
| *Abuadas et al 2018*  **Jordan**  (Amman) | Educational intervention based on the Health Belief Model | Increasing CRC incidence and no screening programme for average-risk individuals. | 1 x FOBT | **Design:** Quasi-experimental study with control group  *Based on Health Belief Model*  **a.** 1-hour educational session on CRC and screening recommendations (IG), i.e. presentation, discussion, hand- out of educational materials on CRC and screening recommendations provided to patients visiting the hospital out-patient department  **b.** participants were offered ‘cards’ to perform screening via FOBT  *Test free of charge for participants* | **Recruited & delivered:**  Research team | Face-to-face | University hospital | 1^st^ July –  3^rd^ Nov 2015  1 x 1hour education session  Data was collected 4 weeks later | Adults aged 50-75 y. | Program based on Health Belief Model | Not reported  (Except for measurement tool, i.e. modified Arabic version of the Champion Health Belief Model Scale) |
| *Li, Qian, et al 2019*  **China**  (Pudong New Area, Shanghai) | Large community-based CRC screening programme, invited by post | High CRC incidence and mortality in China.  Aim was to evaluate the quality (performance during implementation) of the programme. | 1 x FOBT,  1 x HRFQ  Colonoscopy if FOBT or HRFQ positive | **Design:** Prospective cohort study    **a.** Screening invitations sent to target population by primary care physicians  **b.** HRFQ and FOBT was provided  **c.** Reminders were sent if no response within 1 month, reminder was sent once a month for 3 months  **d.** FOBT positive participants were referred for colonoscopy  *Test free of charge for participants* | **Recruited & delivered:** Primary care physician | Mail | Community Health Centres | 2 x screening rounds between 2013-2016 | Residents with medical insurance aged 50-74 y. | Primary care physicians at CHC were used to reduce service cost.  Authors conclude that risk assessment may need to be better tailored in future to better assess target population’s risk. | Residents who did not meet the age criteria were screened if interested.  Modifications were made to some measures in the analysis to ensure accurate program performance indicators. |
| *Salimzadeh et al 2013*  **Iran**  (Tehran) | Education intervention based on the preventive health model | High prevalence of CRC in Iran.  Address barriers towards CRC screening to increase uptake. | (not offered as part of intervention) | **Design:** Community-based randomised Trial  *Based on Preventive Health Model*  **a.** Participants were identified from health club registry and contacted by phone  **b.** In-person interviews were conducted with agreed participants  **c.** IG: Face-to-face education with research assistant (20 min to review education booklet)  **d.** 3 min reminder call after 2 weeks (up to three attempts) (to provide encourage screening attendance)  **e.** Screening was self-reported (either FOBT or colonoscopy)  *Participants covered potential screening costs* | **Recruited & delivered:** Researchers | Telephone  Face-to-face | Community health clubs | July 2011-Nov 2012  1 x education session  1 x reminder call | Adults aged ≥50 y. | Interviews in health clubs (convenience)  Theoretical constructs were incorporated and tailored to Iran. | Not reported |
| *Huang et al 2011*  **China** (Shanghai - 1 community from each of 4 districts) | Community-based health education intervention | High CRC incidence and mortality in China. Community education may be important for screening uptake. | 1 x FOBT | **Design:** Quasi-experimental  **a.** Recruitment: door-to-door to attend lectures  **b.** Monthly lectures in community centres after which participants received information leaflets and FOBT kit  **c.** Face-to-face interview post lecture  *Test free of charge for participants* | **Recruited & delivered:**  Health workers from CDC & public hospitals | Face-to-face | Community/ local community health service centres & Centre for Disease Control | May 2008 – May 2010  Monthly lectures | Local residents | Lectures conducted in local community recreation centres | Not reported |
| *Lin et al 2019*  **China**  (Guangzhou) | Mass media and community intervention | The screening protocol has been adapted by China National Committee of Cancer Early Detection and Treatment to address increasing CRC incidence. This study evaluated compliance and yield. | 1 x HRFQ  2 x FIT  Colonoscopy if HRFQ or FIT positive | **Design:** Cross-sectional  **a.** Newspapers & TV reported that government is inviting all residence to CRC screening  **b.** Everyone eligible received SMS reminder  **c.** HRFQ completed by GP in local CHCs  **d.** 2 x FIT screens (repeated after one week) - faecal samples collected by CHW in health centres  **e.** Referral for colonoscopy if FIT positive  *Test free of charge for participants* | **Recruited:** Media  **Delivered:**  GP & CHW  *(Municipal government provided subsidies)* | Mass media, text message  Face to face | Community Health Centres | 2015-2017 | Residents aged 50-74 y. | Stool collection by CHW to improve participation | Not reported |
| **Colonoscopy only** | | | | | | | | | | | |
| *Chen et al 2019*  **China**  (22 cities in 16 provinces) | Population based screening of high-risk individuals | Lack of evidence on participation and diagnostic yield of population-based CRC screening by colonoscopy in China. | 1 x HRFQ  Colonoscopy if positive | **Design:** Cross-sectional  **a.** Participants were invited by phone calls and personal encounter with trained staff  **b.** Social media and community advertisements were used to raise awareness about programme  **c.** HRFQ assessment by trained staff  **d.** Colonoscopy if HRFQ positive  *Test free of charge for participants* | **Recruited & delivered:**  Trained staff | Face-to-face  Phone calls | Community | October 2012- October 2015 | Residents aged 40-69 y. | Risk assessment was based on Harvard Risk Score but was tailored to Chinese population | Not reported |
| *Garcia-Osogobio et al 2015*  **Mexico**  (Medica Sur Hospital) | Workplace screening intervention | Data collected on CRC incidence and mortality in Mexico is poor. Aim was to determine the prevalence of CRC neoplasm. | 1 x Colonoscopy | **Design:** Cross-sectional  **a.** Recruitment: Personalised invitation letter sent to target population & public announcements were posted at hospital website  **b.** Informative meetings about study/ screening  **c.** Personal interviews were conducted to ensure participants were asymptomatic  **d.** Colonoscopy was conducted with all eligible participants  *Test free of charge for participants* | **Recruited & delivered:** Employer | Mail  Website  Face-to-face | Workplace (Hospital) | 2009-2010 | Employees aged 40-79 y. | Personalised letters | Not reported |

CHC – community health clinics/ centres; CHW – community health worker; CRC – colorectal cancer; FOBT – Fecal Occult Blood Test ; FIT – Fecal Immunochemical Test; GP – general practitioner; HRFQ – high risk factor questionnaire; HN – health navigator; HW – health worker; y – years

*Where: all colonoscopies were conducted at a hospital
