# Supplementary material for: The implementation of colorectal cancer screening interventions in low-and middle-income countries: a scoping review
Source: BMC Cancer. 2021 Oct 19;21:1125. doi: 10.1186/s12885-021-08809-1 (PMC8524916; doi:10.1186/s12885-021-08809-1)
Supplement: Supplementary file 5 — Additional file 5 Supplementary Table 5. Data extraction implementation measures [file 12885_2021_8809_MOESM5_ESM.docx]

**Supplementary Table 5**. Data extraction implementation measures

| Reference  **Country** | Acceptability | Adoption | Appropriateness | Feasibility^a^ | Fidelity | Implementation cost/ resources/ training | Intervention complexity | Penetration/ linkage with other services | Reach/ Uptake/ Socio-demographics | Sustainability |
| --- | --- | --- | --- | --- | --- | --- | --- | --- | --- | --- |
| **Stool test uptake >65%** | | | | | | | | | | |
| *Cai et al 2011*  *Cai et al 2016*  *Ma et al 2012*  **China** | Not reported | Government programme. Study followed revised screening protocol based on previous pilot study. | Use of HRFQ as screening tool is cheap for use in resource-poor settings.  Jiashan country was identified as an economically and medically underserved community but income/ health insurance of participants was not assessed.  Involvement of neighbourhood committees suggest local adaptation, but this was not further described. | Demographic information obtained from local demographic administrative department.  Study population captured with administrative databases for at least 2 years.  Cancer cases were confirmed by Jiashan Cancer Registration System. | Not reported | High-risk factor investigation was completed by well-trained physicians.  Faecal samples were tested by experienced technicians.  At least 2 independent pathologists examined positive pathologic slides of positive lesions.  Coverage of transportation fees were mentioned & breakdown of costs are given. | Multiple steps and stakeholders involved who went into community to complete HRFQ & FIT (timely). | All eligible residents from included communities were invited.  *Ma et al:*  28 villages were included – 53.9% of total population met eligibility criteria.  Collaboration with other organisations not mentioned. | Enrolled: n=31,963  HRFQ completion: 84.7%  FIT completion:  76.4%  Colonoscopy completion: 78.7%  Mean age: 53.9 y  (SD 9.5)  Compliance females: 86.1%  Compliance males: 84.1%  *Ma et al (2 rounds of FOBT) completion:*  (x1): 90.1%  (x2): 77.2% | Intervention based on a screening protocol recommended and put forward in 2006 by the China National Committee of Cancer Early Detection and Treatment.  Based on the cost-effectiveness analysis, the authors concluded that mass screening is beneficial in a high-incidence area.  Long-term plans/ funding were not discussed. |
| *Gong et al 2018*  **China** | Not reported | Adopted by 223 CHCs and 50 hospitals | Capacity for colonoscopies was considered and taken into account, i.e. 25% spare capacity was identified, mostly in 2-tier hospitals.  Higher rate uptake of colonoscopy screening in rural areas vs urban (adjusted OR 1.43, 95% CI 1.40-1.47). | The Shanghai CRC screening registry and management system was an internet-based system that was created for the Shanghai CRC screening program. | Some participants may have been lost at follow-up as they chose higher-level (3-tier), non- designated, hospitals for further examination | Health workers (n=773) from CHC (GPs, nurses, staff from district CDCs and physicians from hospitals) were recruited for training.  Municipal Center for Disease Control and Prevention organized specialists from surgery, endoscopy, and pathology to ensure quality of the diagnosis and treatment of CRCs and lesions.  Training was provided annually. | Complex intervention including a media campaign & involvement of numerous stakeholders and neighbour-hood committees. | All eligible residents from included communities were invited.  Shanghai Center for Disease Control  and Prevention was responsible for program management, data management, training, quality control, and program evaluation. | Participants targeted: 1 million  Participants registered: 828,302  HRFQ & FIT completion: 809,528 (97.7%) with  Colonoscopy completion: 39.8%  Female: 60.9%  Male: 39.1% | This study was part of the CRC screening approach outlined by the Shanghai Municipal Commission of Health and Family  Planning, Shanghai Municipal Finance Bureau, and Shanghai  Municipal Human Resources and Social Security Bureau. |
| *Zheng et al 2003*  **China** | Not reported | Intention was to optimize the mass screening protocol and evaluate its efficacy in low-incidence areas where mortality reduction by FOBT has not been demonstrated  Stool test was especially developed for programme | Not reported | Residents were identified from the complete housing-book registry system.  Study used data recorded on cancer registry system | Not reported | Not reported | Seems to be a complex intervention due to the nature of the mass media approach, although there is a lack of information about participant recruitment. | All eligible residents from the target area were enrolled (10 townships).  Collaboration with other organisations not reported. | Participants recruited n=75,813 (out of 192,261 eligible residents,  39.4%)  RPHA-FOBT & questionnaire completion:  82.7 %  Sigmoidoscopy completion: 73.6%  Socio-demographics not report | Programme conducted over 7 years suggests long-term sustainability, although future funding not reported. |
| *Hassan et al 2016*  **Malaysia** | Not reported | Not reported | Author state that a strengthening of program is needed to achieve a better detection rate  Kota Setar patient enrolment (62.3%) vs Kuala Muda (37.7%) | Study was using secondary data. Data management from primary study not reported. | Author note that there might be missing data on iFOBT collection.  78.2% participants were recruited from clinics, 27.2% recruited from hospitals | Not reported | Complex intervention -several stages involved. Relying on patient to come back for counselling/ repeated screening.  Data collection on compliance potentially tricky/ incomplete. | Initiative was under MoH, no other collaborators mentioned. | Subjects enrolled: n=750  iFOBT compliance 1^st^ round: 94.7%;  2^nd^ round: 90.6%  Colonoscopy completion: 68.1%  61.3% Malay, 31.7% Chinese, 6.5% Indian  Female: 52.3% | Not reported |
| *Noriah et al 2010*  **Malaysia** | Authors described patient barriers for FOBT screening include  being unsure of taking the test, feeling well,  having no symptoms and thinking that they  understand colorectal cancer. | Not reported | The Chinese ethnic group was overrepresented | Not reported | Residents: approached n=2,574  N=605 agreed to participated (24%)  Minimum sample size for house-to-house: n=168; campaign: n=1784  Opportunistic: n=382 | The awareness campaign was the cheapest approach, followed by opportunistic testing & house-to-house approach.  Training and quality assurance not reported. | Different levels of complexity based on approach. | Intervention was run by the Ministry of Health. Collaboration with other organisations not reported. | Agreed to participate by approach:  House-to-house: 86.6%  Opportunistic: 44.8%  Awareness campaign: 13.8%  FOBT completion (91%) by approach:  House-to-house: 95.4%  Opportunistic: 92.2% Awareness campaign: 87.6%  Female: 51.4%  50-59 y: 51.2%  60-69 y: 34.9  Malay: 42.5%,  Chinese: 37.9% | Authors recommend to roll out opportunistic. Long-term plans/ funding not reported. |
| *Tze et al 2016*  **Malaysia** | 4/6 diagnosed CRC patients followed through with all recommended treatments | Inclusion of community leaders and delivery of  door-to-door exercise in 3 local languages | 48.1% of those recruited had an income <RM 1000 and were classified as low-income | Demographic surveys had to be completed by project team to identify number of people at risk within pre-selected communities.  Data management was not reported | Screening was conducted over 5y. as intended.  Unclear how many participants were targeted/ approached | Four training workshops for student volunteers.  Quality assurance was not reported. | Complex due to reliance on volunteers, community leaders, intensive recruitment and emotional and moral support for diagnosed patients. | Charity collaborated with welfare, medical & paramedical organisations (governmental and non-governmental) grass root & community leaders. | Agreed to complete FIT between 2010-2015: n=1581  FIT completion: 80.0% (varied between 80-100% over 5 y.)  Colonoscopy completion: 70.3% (varied between 63.2%-78.6% over 5 y., highest in 1st y.  Female: 60.5%  Malay: 51.2%  50-59y: 44.2%  60-69y: 41.1% | Although this was a 5 year programme, and governmental organisations were involved, reliance on volunteers and lack of further funding reported suggests lack of long-term sustainability. |
| *Aniwan et al 2017*  **Thailand** | 25 subjects missed the stool collection and 15 subjects had poor bowel preparation. | 6 hospitals participated  Study is assessing optimal FIT cut-off point to adopt for future research.  Use of Asian Pacific Colorectal Screening scoring system has previously been validated. | 1222 average-risk and 491 high-risk individuals were identified.  Participants may be biased as based on self-referral | Data management was not reported | Minimum of 1,700 participants were set out to be recruited. This target was met. It seems that the screening was delivered as planned.  6 clinics were involved as set out at the start. | No resources needed for recruitment.  Trained health care staff were recruited/ no other training mentioned.  Programme quality assurance not reported. | Limited recruitment activity (i.e. patients who came to hospitals for other reasons were recruited). | 6 hospitals across Thailand were involved.  No mention of the inclusion of other organisations. | Not reported how many patients were approached.  1713 recruited out of 1740 enrolled (98.4%). | Not reported |
| *Remes-Troche et al 2020*  **Mexico,** Veracruz | 11 participants did not sample stool correctly | Publically funded insurance started to cover CRC treatment for 85% of population in 2015 but no CRC screening programme is in place | Not reported | Data management was not reported | Not described, flow-chart and methods suggest following of programme. | Not reported | Moderate complexity as recruited through newspaper advertisement but one-to-one interviews are time intensive | One laboratory was used as hub for FIT testing.  No link with other services described | Replied to advertisement: n=502  Participants eligible n=473  FIT completion: 85.8%  Colonoscopy completion: 87.5%  Mean age: 61.3 y (SD 7.6)  Male: 31.7% | Authors reported that limited endoscopy capacity could result in lack of program sustainability |
| *Dimova et al 2015*  **Bulgaria** (Plovdiv) | 78.8% participants returned questionnaire  21.2% dropped out after being handed the kit.   ≥90% of participants were satisfied with info received by GP, did not have difficulties carrying out the test, did not need help to perform or interpret the test  10% needed additional discussion on how to use the kit | Reported that intervention was based on evidence that provided the basis for the task force recommendation  Information was delivered in dual language (English & Bulgarian). | n= 5 had ‘difficulty with breaking the  top of the collection tube’ and n=5 had ‘difficulty  with taking stools sample’  3.2% encountered difficulties carrying out the iFOBT; 5.9% needed help in performing the test and 7.6% needed help interpreting of the test  More difficulties were encountered by those aged >70y and those with lower education levels | GPs were selected from National Health Insurance Fund website  Participants completed questionnaire and reported back to GP. No other data storage/ linkage described | Aimed to recruit 30 GPs and 20 patients each which was achieved (unclear how many GPs/ participants were contacted before number was reached) | No lab required as patients analysed samples at home and reported back to GP.  GPs were only asked to recruit 20 participants each – little time investment per clinic | Straight forward as participants were asked to self-complete and analyse test. Individuals were contacted and identified by GPs (time intensive if this was to be rolled out to more patients) | 30 GPs were involved from one region. | Invited n=600  Participated: n=463 (78.8%)  Colonoscopy completion: 75%  Female: 54.7%  Secondary education: 53.7%  Tertiary education or above: 34%  Mean age 61 y (SD 10.29)  24 GPs were female, 17 urban | Opportunistic screening was discontinued nationwide in 2009 although authors recommended to roll out programme |
| *Sucevaeanu et al 2005*  **Romania**  (Dobrogea) | 29.6% of patients interested in screening found diet too restrictive or colonoscopy too risky and did not receive screening | Not reported | Not reported | Data management was not reported | Not reported (seems that everything took place as per protocol) | Already trained GPs delivered the intervention. Further resources not reported. | Moderate complexity as patients recruited through newspaper ads and then further interviewed by GP (time-consuming) | Intervention was conducted in one hospital, delivered by 12 GPs  Linkage with other services was not described. | Patients interested n=1769  Participation: n=1245 (70.3%)  Mean age: 60.2 y (SD 7.5)  Male: 57.5% | Authors concluded that patient recruitment can be improved as this may influence the number of people agreeing to participate and programme sustainability. |
| *Scepanovic et al 2017*  **Serbia**  (Population-based) | Not reported | Take-home FIT was suggested by primary care physicians  Previous postal intervention in Serbia had low uptake, therefore a different approach was tested. | Participation varied between region (46% -100%) | Mention of ‘database’ that held information but no linkage of other databases described | Not described (although seems implemented as intended)  Colonoscopy was delivered within 37 days (close to 31 days according to European guidelines 2008) | Test was processed by trained staff. No other training was described.  Patients were recruited during clinic visits – limits resources invested in recruitment. | Complex population-based intervention but recruitment through GPs seems straight-forward | 50 primary healthcare centres (1/3 of primary healthcare facilities) from 25 administrative regions participated.  50,895 people (7.3%) of target population were invited to participate. | Participation: 87.3%  FIT completion: 77.6%  Colonoscopy completion: 69.7%  Socio-demographics not described | Nation-wide programme suggests it can be run at a large scale but long-term sustainability was not tested.  Authors report a shortage of colonoscopists, which may lead to a delay if there is a large demand. |
| *Gholampour et al 2018*  **Iran**  **(**Fasa City) | Not reported | Informed by the Health Belief Model  Intervention seems very personalised to address individual barriers | Post-intervention the IG significantly improved on all measures assessing health beliefs and CRC awareness compared to pre-intervention. There was no significant improvement in the CG. | All data was self-reported (twice) – seems that screening data was obtained from hospital/ clinic | Number of participants were recruited as per sample size calculation.  Unclear how many participants attended each session | Researchers, clinics, laboratory staff were involved in intervention.  Interviewers were trained.  Time/staff intensive intervention. | Complex education intervention (numerous lectures, counselling, involvement of family etc.) | 2/6 health centres in Fasa city were involved. | Participants: n=200  Not clear how many sessions were attended by participants  FOBT completion  IG: 74%  CG: 6%  Male: 100%  Mean age  IG: 63.18 (SD 8.25),  CG: 65.11 (SD 7.66) | Intensive education intervention with several layers – does not seem sustainable at a large scale |
| *Salimzadeh et al 2017*  **Iran**  (Tehran, rural & urban) | Recruitment over phone: 96.3% and home visits: 3.7% | Informed by Digestive Diseases Research Institute and health care staff  Individualised programme as participant’s  concerns were addressed in lay language by health navigators. | Greater uptake of FIT in rural areas | Primary health care database or municipal registries were used to identify & invite participants | It seems that all 6 health centres participated (although not reported) | HN were hired and trained to deliver intervention.  HN save costs (instead of doctor consultation).  Lab staff were trained. | Straight forward intervention, although individual approach is time consuming. | 3 rural and 3 urban health care centres were randomly selected from study area. | Invited: n=1542 (n=104 not eligible)  FIT completion: 96%  82.4% of FIT + participants could be reached and were invited for colonoscopy  Colonoscopy completion: 60%  Female: 62.9%  Mean age 54.1 (SD 7.0)  Medical insurance: 95.5% | Not reported although authors highlight limited number of trained endoscopists which might limit roll-out |
| **Stool test uptake 45-65%** | | | | | | | | | | |
| *Khuhaprema et al 2014*  **Thailand** | Not reported | All 154 PCUs and 12 CHs (100%) in the province participated in the project. | Urban districts mainly reached through posters vs face-to-face in rural areas, likely led to higher participation amongst rural vs urban districts (73.2%, vs 45.1%).  Higher participation in those aged 60-65 y. vs 50-54 y. (78.9% vs 52.9%). | Forms were specifically developed and entered in a multiuser programme database with inbuilt validation checks  Linkage with database and cancer registry enabled. | All 154 primary care units and 12 community hospitals participated.  No explicit fidelity check was reported.  Monitoring and quality assurance procedures indicated fidelity to the screening plan | Existing resources were used (i.e. no extra staff was hired, no additional pay provided).  One-day training on information dissemination and motivation of target population in CRC screening was provided.  Quality assurance was put into place | Complex intervention involving a large number of clinics and hospital; robust monitoring was put into place. | All eligible participants from the district were invited to participate. All PCUs and CHs from the district participated.  NCI Bangkok and provincial health authorities developed programme jointly with IARC. | Invited:  N= 127,301  iFOBT completion:  62.9%  Colonoscopy completion: 71.8%  Mean age Female:  56.6 y.  (SD=4.3)  Male: 56.8 y. (SD=4.3) | Cost-effectiveness analysis will be determined based on findings to estimate funding required for nationwide programme.  Long-term data lacking – plan for follow-up every 5 y. |
| *Bankovic Lazarevic et al 2016*  **Serbia**  (Population-wide) | Coverage by invitation and participation rates were higher in 2014 compared to 2013. | Programme took lessons from European Guidelines for Quality Assurance of CRC screening (2010)  Personal letters/ phone call as invitation | National screening programme - appropriateness not described but several major institutes involved in the running of the programme and primary care physicians/ local hospitals  Participation rates varied a lot between the municipalities | Health insurance database was used to identify eligible participants | Aimed at recruiting 75% of target population and 19% of total target population were invited | Primary clinics conducted screening within their available capacities which did not affect primary occupation – suggests that no new resources (other than test) were needed | Complex population-based intervention as participants were contacted individually, and several hospitals, clinics and labs were involved to deliver programme | 19% of target population was invited.  Programme collaborators: Ministry of Health, Republican Expert Commission  for Implementation of the Program for Early Detection  of Malignant Diseases in the Republic of Serbia, Republican  Fund for Health Insurance, Cancer Screening Office and local  health institutions (network of 24 public health institutes in each district) | FIT completion: 62.5%  Colonoscopy completion rate: 42.1%  Socio-demographics not described | This was a national organised CRC screening programme that took place over 2 years and authors discuss ‘forthcoming rounds’ indicating future screening |
| *Huang et al 2014*  **China** | Not reported | Authors are suggesting a revised government screening programme based on this study (mainly concerning the follow-up of patients with positive colonoscopy and without removal of polyps) | Not reported | Secondary data was used from the ‘‘Comparison and  Evaluation of Screening Programs for Colorectal Cancer in Urban  Communities in China’’ programme  Programme required linkage with cancer registry | Not reported | Cost of different screening scenarios were compared. Highest cost for FOBT & HRFQ combined, lowest cost for FOBT only as initial screening tool.  Since secondary data was used, quality assurance and training was not described. | Unclear due to lack of information reported. Likely FOBT + HRFQ are more complex than FOBT alone. | Not reported | Patient compliance with colonoscopy after FOBT or FOBT + HRFQ positive test was 37.3% vs 46.8% | Authors suggest a more cost-effective solution as an alternative to the current practice, which is likely more sustainable if supported by the government. |
| **Stool test uptake <45%** | | | | | | | | | | |
| *Wu et al 2019*  **China** | Not reported | Aim was to optimize the risk assessment tool and seek an optimal initial  screening protocol for CRC in this population  Risk score was modified to improve the ‘fit’ for the population. | Number of participants from with middle or occupation school education 58.3%  10% of participants were stratified as high-risk | Data linkage with Shanghai Cancer Registry  Authors reported that all the information are easy to collect, ensuring feasibility of the system in the ‘real world’. | Not reported | Intensive quality control in place (i.e. supervised data collection and data entry, double-checking of data entered to improve quality).  Annual training for physicians | Complex intervention including data record linkage and area of coverage.  One-to-one interviews at screening stage likely burdensome at population level. | All eligible residents from the target area who had basic medical insurance were invited. | Eligible participants: n=1,356,068  Screening completion: 39.7%  Colonoscopy completion: 23.5%  Female: 59.2%  Aged 60-69 y.: 53.8% | Duration of the study (2013-2017) suggests long-term sustainability as service is offered by regular health clinics.  Planning for a third round of screening was in place (2017-2019). |
| *Abuadas et al 2018*  **Jordan**  (Amman) | Not reported | Informed by the Health Belief Model  Intervention was delivered in a well-lit room with comfortable chairs | Post-intervention: IG participants showed significantly higher levels of knowledge, perceived susceptibility and perceived severity and fewer perceived barriers compared with the CG  Likely selection bias and ~30% in each group know someone with CRC | All data was self-reported.  Data management was not reported | No required sample size was mentioned prior to intervention  Intervention seems delivered as intended | Existing staff were asked to deliver intervention.  No training/ cost was described. | Intervention straight- forward (convenience sampling and education only, screening attendance self-reported) | Participants recruited from two hospitals.  Referral to laboratory for screening if participants were interested. | Participants n=197  Intervention completion  IG n=98/121 vs CG n=99/121  Higher screening in IG vs CG (35.7% vs 8.1%)  Male: 50.8%  Mean age: 59.1y (SD 7.4)  Secondary education: 50% | Not reported  Nurses/ physicians were the least reported source of information for CRC – implementing a HCP led intervention would require HCP to free up time |
| *Li, Qian, et al 2019*  **China** | Not reported | Authors suggest that intervention aligns with local environment but don’t describe how. | Participation rate:  Urban (20.3%)  Suburban (58.80%)  Rural (62.11%)  8.42% of tested participants were outside of the target age range  Referrals to patients were sent based on travel distance to closest hospital. | Established data reporting system to follow-up participants.  Behaviours and results observed during each step were submitted to the program reporting system. | 29.9% of target population were screened which was lower than the goal of screening 21% (n=218,489 participants) in 1^st^ round and 7% in each of the three years for the second screening (42% in total  screened -lower than goal). | Quality control in place (monthly data verification, annual onsite oversight, annual review of data entry accuracy and timeliness as well as progress for each community health centre)  Training organised annually for physicians.  Physicians were paid subsidies (incentives were associated with the quality control outcomes). | Complex intervention based on area to cover, data reporting and quality assurance measures put in place. | All eligible residents who had basic medical insurance from included communities were invited.  The program established a total of 13 one-to-more screening collaborations  between designated hospitals and CHCs. | Invitations sent n=1,262,214  Screening completion:  35.2%  Male: 41.27%  Female: 58.73%  Aged 50-59 y.: 15.48%  Aged 60-74 y.: 65.54%  Out of target age range (8.42%)  *Higher reach to those ≥ 60 y. due to other public health programme recruiting participants at that age running at the same time* | Program was launched & paid for by the government as part of essential public health service.  Funds were distributed to each of the 46 communities within district. |
| *Salimzadeh et al 2014*  **Iran**  (Tehran) | Not reported | Intervention was informed by preventative health model | Knowledge improved in IG compared to CG, however, low screening uptake suggests appropriateness can be improved | Limited administrative data (self-reported) | Seems that intervention was implemented as inteded - 47 people lost at follow up (10 from IG and 37 from CG) | Health club workers received a 6-hour training to act as research assistants. | Straight forward intervention with delivery of education, respondents were asked to seek out screening themselves | 12 /20 H  health clubs participated.  Linkage with other services not described. | N=360 participants  Screening completion: 26%  Colonoscopy completion: 5% | Not reported, although could be sustained through health-clubs as no medical staff is needed (however, limited uptake) |
| *Huang et al 2011*  **China** | Evaluation score for instructor: 3.08/4  Satisfaction of content: 3.01/4  Satisfaction of administration: 2.99/4  (all slightly higher in Y1 vs Y2) | Not reported | Districts were chosen based on best performance of routine health education according to the administrative report in the local district (may have introduced bias).  High-school education: 38.4% | Screening behaviour was self-reported (no screening records accessed) | Average lecture attendance  Y1: 3.25 lectures  Y2: 2.71 lectures  Not stated how many of the lectures were delivered as intended. It seems that monthly lectures were provided as set out from the start. | Unclear what training health workers received who delivered the intervention. | Door-to-door recruitment of lecture attendees seems timely.  Intervention (i.e. health education) was straight forward.  Data collection was timely (i.e. individual interviews). | One community was selected from each of the 4 districts as the intervention site.  Lectures were provided monthly.  Collaboration with other organisations not reported. | 8,981 lecture participants  n=1041/1240 of those who were recruited to complete the survey participated (96.4%)  FOBT completion: 24.5%  Colonoscopy completion: 12%  Willingness to receive FOBT after Y1: 70%, Y2: 47%  Willingness to receive colonoscopy after Y1: 47%, Y2: 40%  Knowledge and attitude prior the programme were not assessed (limits results)  Female: 61.6%  Age <50y.: 39.5% | Intervention reach was less in Y2, suggesting limited long-term effectiveness. |
| *Lin et al 2019*  **China** | Not reported | First round of mass CRC screening was implemented according to local expert advice after many hearings and discussions. | Authors note that many residents may not have been reached through recruitment channels, hence the low uptake.  Participation from urban (15.1%) and rural (15.9%) areas | Demographic data received from municipal data registry  Specialised online system for CRC screening was established for programme. | Not reported | Quality of data was checked.  Training not described. | Complex mass screening intervention based on promotion, recruitment, reminder and screening efforts (x2). | Mass screening programme of all eligible residents from target area.  Run by the city authorities and supported by the Department of Health. | Eligible subjects: n=2,283,214  Total attendance: 15.4%  FIT completion: 14.0%  Colonoscopy completion: 18.9%  Compliance: males: 11.5%; Compliance females: 19.2%  *Participation rate increased with age.* | Programme was run by city authorities (better chance of long-term sustainability if further supported), however, low uptake suggests programme needs to be improved to be effective and sustainable long-term. |
| **Colonoscopy only** | | | | | | | | | | |
| *Garcia-Osogobio et al 2015*  **Mexico**  (Medica Sur Hospital) | Not reported | Letters were personalised  Use of existing communication channels (employer) | Primary screening with colonoscopy is invasive – likely explains low uptake | Data management was not reported  Demand on provider high (due to personal interviews and screening with colonoscopy) | Conducted in one workplace/ hospital as planned | Colonoscopy requires more resources than a stool test.  Cost covered by employer (hospital).  No extra training needed | Moderate complexity as organised through employer and existing communication channels | Not reported | Letters sent: n=600  Respondents: n=123 (20.5%)  Screening completion: 16.5%  Female: 74%  Male: 26%  Mean age: 50.1 y (SD 7.4) | Not reported |
| *Chen et al 2019*  **China** | Authors concluded that low colonoscopy uptake suggest that non-invasive tests (e.g. FOBT) should be used for screening. | Risk factor questionnaire was adapted for the Chinese population. | Participation rates for colonoscopy screening among high-risk population was very low | Data management system held  all patient information.  Data were transmitted to Central Data Management Team in the National Cancer Centre China. | Not reported | Trained staff, pathologists, gastroenterologists physicians.  Trained study staff checked validity of data entered. | Multiple steps and stakeholders to recruit community and screen participants | All eligible residents from included communities were invited. | Recruited: n=1,381,561  High-risk: n=182,927  Colonoscopy completion: (14.0%, 95% CI 13.8% to 14.2%)  Mean age: 54.7 y (SD=7.8)  Female: 56.1% | Study was part of an ongoing national cancer screening programme. |

^a^ Under feasibility, we mainly reported use of administrative data

CRC – colorectal cancer, SD – standard deviation, y - years
